# Discovery and Characterization of Novel Non-Hydroxamate HDAC11 Inhibitors

**DOI:** 10.3390/ijms26135950

**Published:** 2025-06-20

**Authors:** Aleksandra Kopranovic, Franz-Josef Meyer-Almes

**Affiliations:** 1 Department of Chemical Engineering and Biotechnologiy, Darmstadt University of Applied Sciences, Haardtring 100, 64295 Darmstadt, Germany; 2 European University of Technology, European Union, 64295 Darmstadt, Germany

**Keywords:** HDAC inhibitors, high-throughput screening, non-hydroxamate inhibitors, binding mode, molecular docking

## Abstract

Histone deacetylase 11 (HDAC11), the sole member of class IV HDACs, has gained prominence due to its unique enzymatic profile and pathological relevance in cancer, neurodegenerative, inflammatory diseases, and metabolic disorders. However, only a limited number of selective HDAC11 inhibitors have been identified, and many of these contain a potentially mutagenic hydroxamic acid as a zinc-chelating motif. Consequently, there is an imperative to identify potent and selective non-hydroxamate HDAC11 inhibitors with improved physicochemical properties. In this study, we conducted an extensive experimental high-throughput screening of 10,281 structurally diverse compounds to identify novel HDAC11 inhibitors. Two promising candidates, caffeic acid phenethyl ester (CAPE) and compound 9SPC045H03, both lacking a hydroxamic acid warhead, were discovered, showing micromolar inhibitory potency (IC_50_ = 1.5 and 2.3 µM, respectively), fast and reversible binding, and remarkable isozyme selectivity. Molecular docking revealed distinct zinc-chelating mechanisms involving either carbonyl oxygen (CAPE) or pyridine nitrogen (9SPC045H03), in contrast to canonical hydroxamates. Both compounds are drug-like and exhibit favorable physicochemical and pharmacokinetic profiles, particularly beneficial water solubility and good adsorption, making them valuable starting points for further optimization. These findings open new avenues for the development of selective, non-hydroxamate HDAC11 inhibitors with potential therapeutic applications.

## 1. Introduction

**Figure 1:**
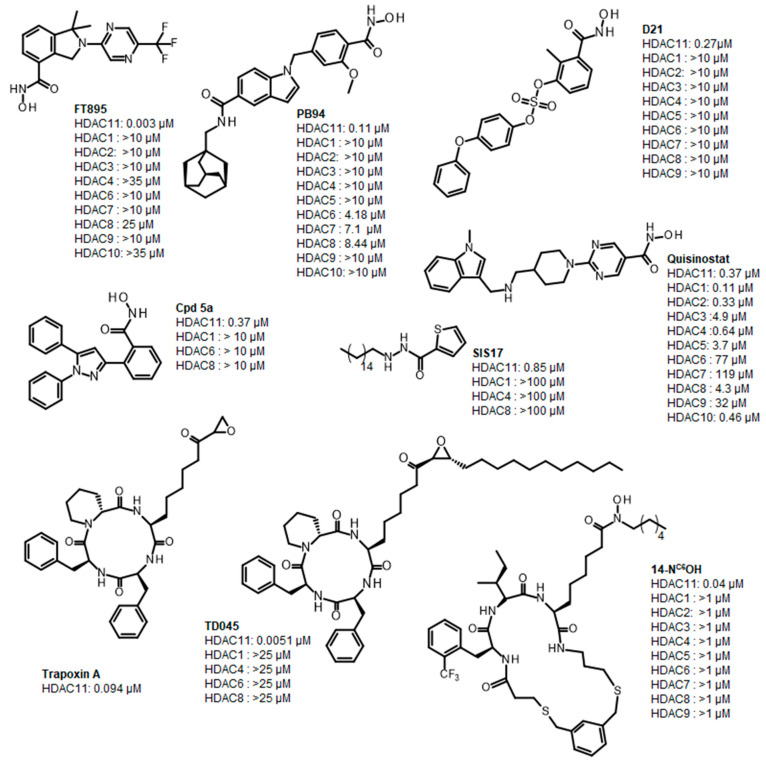
Known HDAC11 inhibitors.

Histone deacetylase 11 (HDAC11) is the sole member of the class IV histone deacetylases, characterized by its unique structural and enzymatic properties. It plays a crucial role in regulating gene expression by removing acetyl groups from histones and non-histone proteins, thereby influencing chromatin remodeling and cellular processes such as proliferation, differentiation, and immune responses. HDAC11 is predominantly expressed in the brain, testis, and immune cells, highlighting its functional significance in neural and immune regulation. The dysregulation of HDAC11 has been implicated in various pathological conditions, including cancer, neurodegenerative disorders, and immune-related diseases. The overexpression of HDAC11 has been observed in several malignancies, where it promotes tumor progression by repressing tumor suppressor genes and modulating immune evasion mechanisms. In neurological disorders, HDAC11 contributes to neuronal dysfunction and cognitive decline by affecting synaptic plasticity and inflammatory pathways. Additionally, HDAC11 has been linked to metabolic disorders and inflammatory diseases, making it a promising therapeutic target in these indication areas. Given HDAC11’s prominent roles in neural, immune, and metabolic regulation—and its implication in a wide range of diseases including cancer, neurodegeneration, and inflammatory disorders—there is growing interest in targeting this enzyme pharmacologically. The pathological relevance of HDAC11 has spurred efforts to develop selective inhibitors that could modulate its activity and potentially restore normal cellular function in these disease contexts. Consequently, several small molecules have been identified as HDAC11 inhibitors, although only a limited number combine both high potency and isoenzyme selectivity. Among these are a handful of hydroxamic acids, some of them showing considerable selectivity against other zinc-dependent HDAC isoenzymes (Figure 1). The class of HDAC11 inhibitors with a hydroxamate warhead includes quisinostat [1], ortho-methyl benzene hydroxamic acids from the D-series, for example, D21 [2], Cpd 5a [3], and probably the best HDAC11 inhibitor in terms of potency, selectivity, and physicochemical properties, FT895 [4]. Another important reference substance in many HDAC11 publications is the alkyl hydrazide SIS17, although a very long alkyl chain with 16 C atoms severely limits the solubility of the substance [5]. Based on the cyclic peptide inhibitor trapoxin A, which is a non-selective HDAC11 inhibitor, analogs with improved potency and selectivity have been developed. The best representatives of this class of cyclic peptides are TD045 [6] and 14-N^C6^OH [7], which show very good isoenzyme selectivity and IC_50_ values of 0.0051 and 0.04 µM, respectively (Figure 1).

14-N^C6^OH carries an N-hexyl-substituted hydroxamic acid that replaces the epoxide in trapoxin A and TD045 as a warhead. Hydroxamic acid-containing substances are known to chelate metal ions, which makes them potent inhibitors of metalloenzymes [8]. Not only are HDACs targeted but also matrix metalloproteinases (MMPs) [9], carbonic anhydrases (CAs) [10], 5-lipoxygenases (5-LOXs) [11], angiotensin-converting enzyme (ACE), a Zn^2+^ dipeptidyl carboxypeptidase [12], and urease [13] to name a few important target classes of hydroxamic acids. In most cases, new HDAC inhibitors are tested on a panel of isoenzymes to determine their selectivity. However, it cannot be excluded that other metalloenzymes are also inhibited, which could possibly lead to undesired side effects. Since hydroxamic acids complex multivalent metal cations, it is also conceivable that they could disrupt their homeostasis in the cell. There is also a well-founded concern that hydroxamic acids are converted into reactive isocyanates in the cell in a Lossen rearrangement-like reaction, which could react covalently with nucleophilic species such as adenine or guanine bases and thus have a mutagenic effect [14]. In fact, all marketed HDACs cause chromosomal aberrations in rodent cells. Therefore, there is a great need for a replacement of the canonical zinc-chelating warhead hydroxamic acid with other functionalities. A major challenge in HDAC11-targeted drug development is achieving selectivity. Many HDAC inhibitors exhibit broad-spectrum activity, affecting multiple HDAC isoforms or other metalloenzymes, which can lead to undesirable off-target effects. The structural similarities among HDAC family members make it particularly difficult to design inhibitors with high specificity for HDAC11, raising concerns about toxicity and adverse reactions. Non-specific HDAC inhibition can disrupt normal physiological processes, leading to unwanted immune suppression, neurotoxicity, and metabolic imbalances. Therefore, the development of highly selective HDAC11 inhibitors remains a crucial goal to minimize systemic toxicity and improve therapeutic efficacy. HDAC11 is very poor at removing acetyl residues from lysine residues, whereas the deacylation of longer fatty acids, such as myristoylic acid, is removed very efficiently. Therefore, many HDAC11 inhibitors contain long alkyl chains that mimic myristoylated lysine residues. These inhibitors, such as SIS17 and TD045, have poor to moderate solubility, which limits their bioavailability. For this reason, the physicochemical properties of HDAC11 inhibitors in particular must be kept in mind from the outset when optimizing the active substances. The fact that known HDAC11 inhibitors often have unfavorable predicted pharmacokinetics and drug-like properties, or carry the hydroxamic acid group as a warhead, clearly indicates that there is a strong need for the development of optimized HDAC11 inhibitors to improve the chances of further development into drug candidates. In this study, we perform an unbiased experimental compound screening to search for new pharmacophores that can serve as a starting point for the development of new HDAC11 inhibitors with improved drug-like properties.

## 2. Results

### 2.1. High-Throughput Screening

Until recently, there was no known crystal structure of HDAC11, partly because of the difficulties associated with purification and the low stability of HDAC11. There is currently an AlphaFold structure of HDAC11, which is quite accurate for the bulk of the enzyme, particularly around the highly conserved active site. However, experimental studies have revealed an extraordinary preference for substrates with lysine residues modified with longer fatty acids rather than acetate. This finding suggested significant deviations from the crystal structures of canonical acetate-removing HDACs. This inspired Sippl et al. to optimize the original AlphaFold structure using computational approaches including flexible docking and molecular dynamics and incorporating experimental knowledge of HDAC11 inhibition by known inhibitors. The optimized AlphaFold structure is certainly an improvement over the original structure, but it is still a predicted structure. Nevertheless, Sippl et al. challenged their optimized AlphaFold HDAC11 model in a virtual screening campaign against a library of more than 12,000 selected chemical structures from the ZINC20 database [15], which is a publicly available database including about two billion small molecule structures [16]. They confirmed one out of five virtual hits to be active against HDAC11, having an IC_50_ value of (3.5 ± 0.5) µM. All hits contained a benzene hydroxamic acid, which is a typical feature of HDAC inhibitors and usually confers most of the binding energy through the chelation of the catalytic zinc-ion in the active site. Notably, all hit compounds had a small ortho-substituent and an extended substituent in the meta position at the benzene hydroxamic acid. Although the experimentally confirmed hit compounds validated the optimized AlphaFold model in some respects, the information about possible conformational flexibility or the existence of more than one HDAC11 conformation in a solution is limited. We therefore decided to set up an experimental high-throughput screening to identify novel pharmacophores that would inhibit HDAC11. Our screening library comprised the LOPAC^®^ library, a collection of 1280 (1277 test set) pharmacologically active compounds with known targets and effects, the SPECS library consisting of 6533 compounds with high chemical diversity, the OTAVA library containing 298 metal-chelating fragments, and our in-house HDA library, which meanwhile contains a collection of 2173 substances synthesized in our group, or from international organic–synthetic partners, a total of 10,281 substances, configured on microtiter plates, with 8 positive and 8 negative controls on each plate.

**Figure 2:**
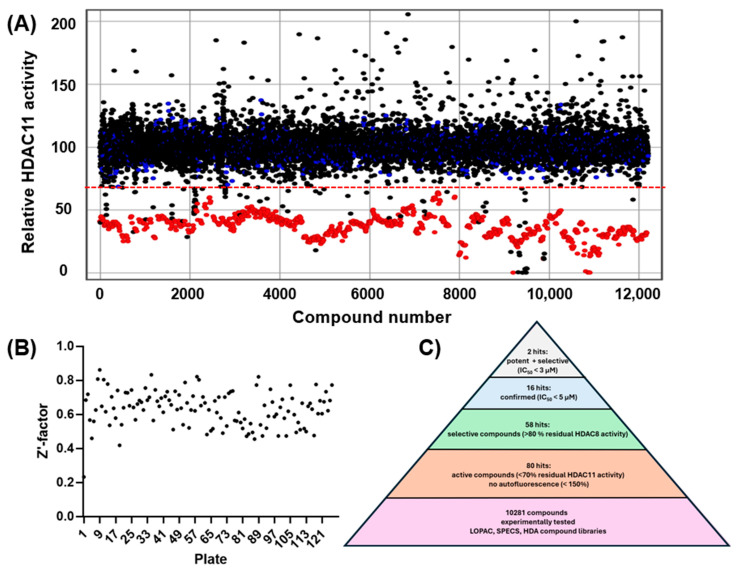
(**A**) Screening of 10,281 compounds against HDAC11. The residual enzyme activity is plotted versus the compound number. Data points for compounds are black, negative (inactive) controls are marked blue, and positive (active) controls are colored red. The dotted red line indicates the hit threshold (<70% residual enzyme activity in the presence of the compound). (**B**) Quality control: calculated z’-factors for every tested microtiter plate. (**C**) Hit pyramid resulting in 2 confirmed potent and selective HDAC11 inhibitors.

**Figure 3:**
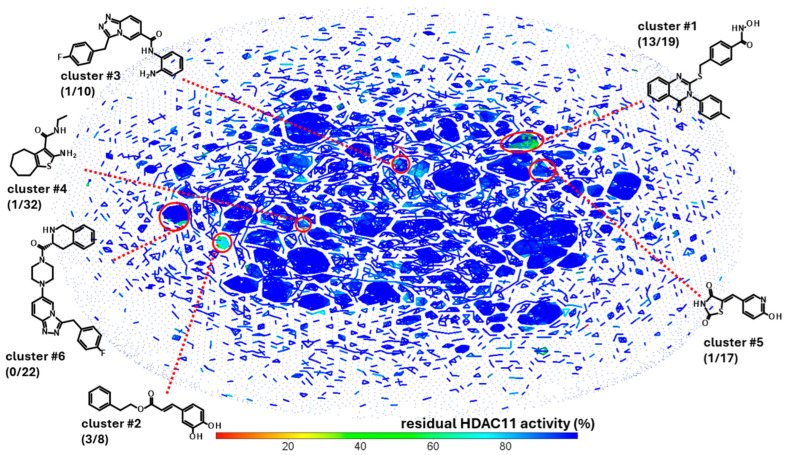
Activity-cliff analysis of 10,281 screened compounds. Compounds were clustered according to structural similarity and colored according to residual HDAC11 activity. Highlighted clusters show compounds with HDAC11 activity, but no or weak activity in a previous counter screen against HDAC8. The number in brackets show the number of compounds with less than 70% residual HDAC11 activity and the total number of cluster members.

The experimental approach was completely unbiased and also offered the possibility of finding compounds that do not contain the problematic hydroxamic acid as the zinc-chelating warhead of typical HDAC inhibitors. HDAC11 was freshly prepared before screening runs over 1–3 days to ensure that a sufficiently active enzyme was used for the screening. The performance of the screen was determined by using high and low controls for HDAC11 activity and calculating the z’-factor for each 96-well microplate. Typically, z’-factors between 0.5 and 0.8 were measured, indicating acceptable to very good performance. A total of 10,281 purified compounds were tested (Figure 2). We observed a low hit rate of about 0.8% and obtained 80 hits showing significant inhibitory activity and little or no autofluorescence (Figure 2C). Using a prior screen against HDAC8, we excluded non-selective compounds that also exhibited activity on HDAC8, resulting in a set of 58 hit compounds. At this early stage, far from clinical application, we did not apply toxicity filters such as PAINS to avoid the potential loss of structurally novel and innovative pharmacophors. Using activity-cliff analysis, we grouped compounds based on structural similarity, revealing six distinct clusters, each containing 8–32 members, where some of them demonstrated significantly higher inhibitory activity compared to negative controls and the majority of other tested compounds. Hit clusters containing multiple active members provide preliminary insights into the structure–activity relationships (SARs) and are of particular interest, as the hits mutually confirm their activity in the primary screen. The number of active compounds with a residual HDAC11 activity of <70% for the hit clusters varies largely from 1 out of 32 (cluster #4) to 13 out of 19 (cluster #1) (Figure 3).

Only clusters with >5 members and at least 1 active member were considered. Also, 12 highly active singleton hits with a residual HDAC11 activity of <50% were added to the list of compounds of interest and retested in the presence of a 35 µM compound (Figure S1). The most noteworthy cluster #1, composed of benzene hydroxamic acids with the quinazolinone cap group, included 13 active compounds out of 19 structurally related molecules. All hits from cluster #1 were confirmed and the dose-response curves were measured.

The most potent HDAC11 inhibitor exhibited an IC_50_ value of 1.1 ± 0.4 µM (Table 1 and Figure S8).

This potency is comparable to the well-known HDAC11-selective inhibitor SIS17 (IC50 = 0.85 µM). In the presence of 50 µM SIS17, the degree of myristoylation of the HDAC11 substrate SHMT2 is significantly increased in cells, indicating intracellular target engagement [5]. Therefore, a similar effect would be expected for our hit compounds with the same cell penetration.

Compounds of hit cluster #2 containing 3,4-dihydroxy cinnamic acid esters and amides were also of high interest, with 3 out of 8 compounds displaying graded IC_50_ values ranging from 1.5 to 14 µM. In contrast, cluster #3 (triazolopyridine carboxamids) and cluster #4 (cycloalkylthiophenes) were deemed less promising due to low confirmed residual HDAC11 activity < 50% in the presence of 35 µM compounds. In cluster #5 only one member, 9HDA018E08, was confirmed with an IC_50_ value of 19 µM indicating low activity against HDAC11, and also in cluster #6, only one compound could be confirmed with very weak affinity (IC_50_ = 66 µM). Among the 12 singleton hits, only one compound, 9SPC045H03, could be confirmed with significant activity showing an IC_50_ value of (2.3 ± 0.5) µM. Cluster #1 was the only hit cluster that contained exclusively hydroxamic acids. This zinc-chelating functionality is present in most academic and clinically approved HDAC inhibitors and confers most of the binding energy. The cluster compounds have been reported before as HDAC6 inhibitors, but nothing was known about HDAC11 activity [17]. Here, we show that the quinazolinone-based compounds are also HDAC11 inhibitors, with the best compound having an IC_50_ value of 1.1 ± 0.4 µM. Unfortunately, the compounds of cluster #1 are also active against several other HDAC isozymes (Table 2). The nature of HDAC–ligand interaction is further analyzed in the following docking section. Caffeic acid phenethyl ester (CAPE, 9LOP004C10) turned out to be the most active compound in cluster #2 with an IC_50_ value of 1.5 ± 0.4 µM. In light of the very low hit rate of the screen, the absence of the common hydroxamate warhead, which is obligatory for most HDAC inhibitors, and the simplicity of the chemical structure, the observed single-digit micromolar activity is quite surprising. In addition, CAPE showed considerable selectivity against other HDAC isozymes. In contrast, the two other HDAC11 active cluster members, the α-cyano-acrylamides Tyrphostin AG 555 (9LOP015D09) and Tyrphostin AG 490 (9LOP015E07), were less potent and more importantly rather unselective (Table 2). An analysis of HDAC11 activities in the presence of compounds structurally similar to CAPE revealed that free caffeic acid or a caffeic acid ester, where the catechol ring was replaced by p-(nitrophenyl)methyleneamino)phenyl moiety (9SPC056H05) that could cause sterical problems, did not inhibit HDAC11 (residual activities 91% and 97%, respectively) (Table S1). Also, hydroxypyran-2-one instead of the carboxyl ester functionality of CAPE (9LOP008H05) led to a loss of activity (82% residual HDAC11 activity). Strikingly, the transition from 3,4-dihydroxy cinnamic acid ester in CAPE to α-cyano-acrylamides (9LOP015D09 and 9LOP015E07) lowered but still resulted in micromolar HDAC11 activities for the best analogs, which was, unlike for CAPE, associated with off-target activities against other HDAC isozymes (Table 2 and Figure S3).

The only confirmed hit compound in cluster #5, 9HDA018E08, has a very low fragment-like molecular weight of 222 Da. Cluster members contained a thiazolidinedione (TZD) group, which could chelate zinc ions. We have extensively analyzed hundreds of TZD analogs for their activities against HDAC isoenzymes and identified several compound series as selective HDAC4 or HDAC8 inhibitors or directed against dual targets of HDACs and VEGFR-2 or PPARγ [18,19,20,21]. However, any modification of 9HDA018E08 caused a dramatic loss in HDAC11 activity (Table S2). Therefore, we see no possibility, even if the starting substance is very small, to further optimize this substance class to potent HDAC11 inhibitors. The best confirmed hit from cluster #6 shows only very weak activity (IC_50_ = >50 µM). For this reason, the optimization of this hit substance will also not be pursued further. The same applies to the singleton substance 9SPC036F04, which shows only a very weak effect against HDAC11 (Table 1 and Figure S8). In contrast, the second singleton hit 9SPC045H03 has very good activity with an IC_50_ value of 2.3 ± 0.5 µM. The 9SPC045H03 compound is more than 30-fold selective for HDAC11 with respect to HDACs 1, 4, and 6, whereas the selectivity against HDAC8 was about 3-fold (Table 2 and Figure S3). The chemical structure of 9SPC045H03 is completely unexpected and different from any previously known HDAC inhibitor. The canonical hydroxamate warhead is missing, which opens opportunities to develop non-hydroxamate HDAC11 inhibitors.

### 2.2. Pharmacokinetics and Drug-Likeness

**Figure 4:**
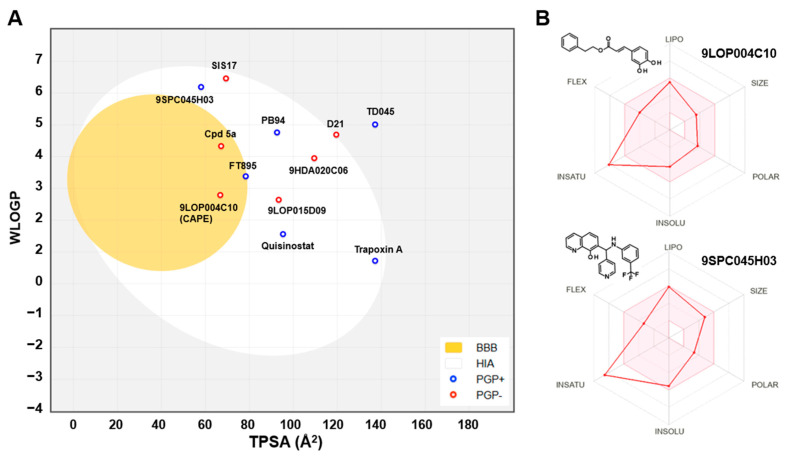
(**A**) Prediction of passive gastrointestinal absorption (HIA) and blood–brain barrier (BBB) permeation in humans for HDAC11 inhibitors using the BOILED egg model implemented in Swiss ADME [31]. The yellow BBB region indicates the region for compounds that passively permeate the BBB. The white region, HIA, contains compounds that are expected to be passively absorbed through the gastrointestinal tract. Blue dots indicate compounds predicted to be effluated from the central nervous system by P-glycoprotein. Compounds with red dots are not expected to be cleared by P-glycoprotein. (**B**) Spider web charts with predicted physical-chemical properties for indicated hit compounds using Swiss ADME.

The hit compounds and known HDAC11 inhibitors were then further analyzed for expected pharmacokinetic properties and structural features important for drug molecules. To this end we used the highly recognized and publicly available web tool Swiss ADME http://www.swissadme.ch/ (accessed on 1 April 2025) to predict the pharmacokinetics, drug-likeness, and accessibility through the medicinal chemistry of potent HDAC11 inhibitors [22] (Figure 4). With the exception of TD045 and D21, all substances are expected to be absorbed by passive gastrointestinal absorption. The hit CAPE is even predicted to penetrate the blood–brain barrier. Looking at the known HDAC11 inhibitors, it is noticeable that many substances, e.g., the cyclopeptides TD045 and 14-N^C6^-OH, and the alkyl-hydrazide SIS17, which are isoenzyme-selective HDAC11 inhibitors, are very poorly soluble in water (Figure S2). This is also reflected in the prediction of drug-likeness, which is not given by most metrices according to Lipinski [23], Ghose [24], Veber [25], Egan [26], and Muegge [27] according to the prediction by Swiss ADME (Figure S2). In contrast, FT895 has by far the best predicted pharmacokinetic and drug-like properties. Quisinostat and trapoxin A are not interesting simply because they non-selectively inhibit other HDAC isoenzymes. The reference substances D21 and Cpd 5a have acceptable pharmacokinetic properties. The corresponding spider web chart is very similar to the hit substances CAPE, 9SPC045H03, 9HDA020C06, and 9LOP015D09 (Figure S2). The limits for a suitable physicochemical range for oral bioavailability are adhered to with regard to lipophilicity (−0.7 < XLOGP3 < +5.0), size (150 g/mol < molecular weight < 500 g/mol), polarity (20 Å^2^ < TPSA < 130 Å^2^), insolubility (−6 < Log S (ESOL) < 0), and flexibility (0 < number of rotatable bonds < 9). Only the lower unsaturation limit is undercut (fraction of sp^3^ hybridized carbon atoms versus total carbon atoms < 0.25), which suggests a highly unsaturated molecule, which might be critical for bioavailability. Nevertheless, the experimental and predicted solubility of these compounds is at least moderate and can be improved by medicinal chemistry methods. The two most potent and selective hit compounds CAPE and 9SPC045H03 have therefore beneficial physicochemical properties, water solubility, and good absorption or even BBB permeation properties, and the compounds are drug-like (Figure S2). CAPE has never been reported as an HDAC inhibitor. But the compound is a well-known inhibitor of NF-κB (nuclear factor kappa-light-chain-enhancer of activated B cells), thereby contributing to anti-inflammatory and anticancer effects [28]. For CAPE multiple other pharmacological effects are reported, such as the inhibition of inducible nitric oxide synthase and cyclooxygenase-2 [29] or the modulation of several pathways such as mTOR and β-catenin signaling [30]. It should be mentioned that there is a PAINS (Pan-Assay Interference Compounds) alert for CAPE, which refers to the contained catechol group. Catechols could oxidize to ortho-quinones, which can undergo redox cycling, generating reactive oxygen species (ROS). This can non-specifically inhibit enzymes through covalent modification.

Catechols can also chelate divalent metal ions such as Zn^2+^. However, CAPE did not show cytotoxic effects (Figure S10), and the observed selectivity for HDAC11 seems to be promising for further investigation (Table 2). The Zn^2+^-chelating properties of CAPE could be used to replace the much more problematic hydroxamic acid moiety of canonical HDAC inhibitors. The optimization of hydroxamic acid-containing HDAC inhibitors has shown that it is possible to increase HDAC isoenzyme selectivity by modifying the head group that interacts with the outer edge of the conserved active site. The small size of CAPE leaves sufficient scope to use CAPE as an interesting starting point for the development of improved selective non-hydroxamate HDAC11 inhibitors. Further optimization using computational and medicinal chemistry methods is currently underway. The second promising hit with respect to activity and selectivity is 9SPC045H03 (Table 2). This compound is also unknown as an HDAC inhibitor. There is only one Chinese patent application, but no literature, which claims that this compound has antitumor activity and would be an inhibitor of the macrophage migration inhibition factor (MIF) (CN105566293 A). There is also a PAINS warning for this substance, which refers to an aromatic Mannich base it contains. Under certain circumstances, such substructures could react covalently with proteins or lead to redox cycling, as described above for catechol. However, these problems can often be solved by specific medicinal chemistry optimizations. We tested the cytotoxicity of 9SPC045H03 against HEK293 normal cells and found a moderately toxic effect (50% cell viability at 100 µM compound concentration). 9SPC045H03 is nevertheless interesting because of its good medicinal-chemical access and unusual structure for an HDAC inhibitor, suggesting novel molecular interactions with HDAC11 that need to be discovered.

### 2.3. Reversibility and Time-Dependence of Binding

**Figure 5:**
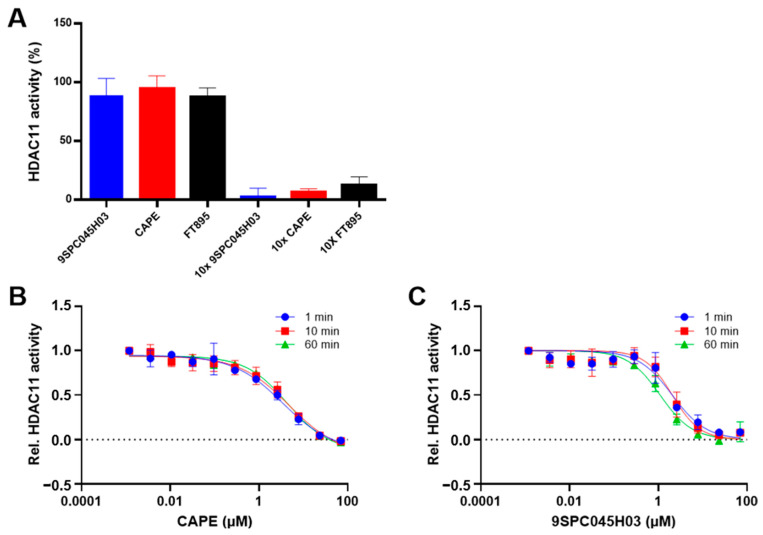
(**A**) Rapid dilution experiment showing the recovery of HDAC11 activity upon dilution (left) compared to almost complete inhibition in the presence of 10 × IC_50_ concentration of the respective inhibitor (right). The data demonstrate the reversibility of the binding of the indicated compounds to HDAC11. FT895, a known fast reversible HDAC11 inhibitor, is used as a control. Time-dependent dose-response curves of CAPE (**B**) and 9SPC045H03 (**C**) against HDAC11, with indicated pre-incubation times demonstrating no time-dependent binding behavior. All data are expressed as means with standard deviations (n = 3).

In the following, we have further investigated the binding mechanism of the non-hydroxamate hits CAPE and 9SPC045H03. To determine the reversibility of binding, a rapid dilution experiment was performed in which a concentrate of HDAC11 and inhibitor, in which the enzyme was almost completely inhibited, was rapidly diluted so that the inhibitor could dissociate from the enzyme. The subsequent recovery of the enzyme activity indicated reversible binding of CAPE and 9SPC045H03 to HDAC11 (Figure 5A and Figure S9).

Next, we investigated the binding kinetics of 9SPC045H03 and CAPE to HDAC11. To this end, we pre-incubated HDAC11 in the presence of compounds for different times up to 60 min and analyzed the inhibition of enzyme activity (Figure 5B and Figure S7) and observed no time-dependent inhibition for CAPE, 9SPC045H03, and the FT895 reference inhibitor. Together, the rapid dilution and time-dependent inhibition experiments show that the binding of CAPE and 9SPC045H03 is fast and reversible.

### 2.4. Molecular Docking

**Figure 6:**
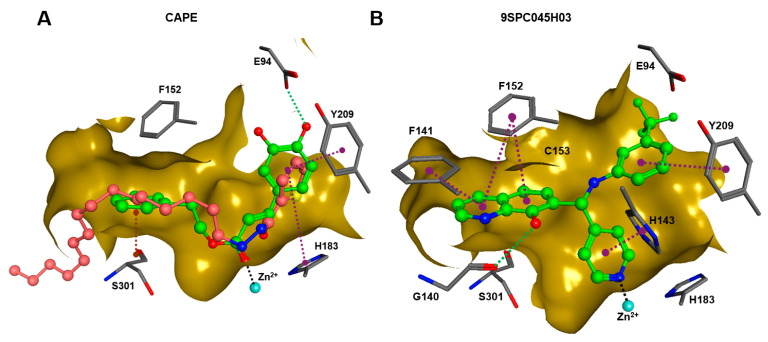
(**A**) The binding pose of CAPE (green) and SIS17 (dark-pink) in the active site of HDAC1, and (**B**) the binding pose of 9SPC045H03 (R-enantiomer) as determined by molecular docking. The dark-yellow surfaces represent the rear surface of the binding pocket; the front part is transparent to allow a view inside. The catalytic zinc ion is shown as the cyan sphere, the green dotted lines indicate conventional hydrogen bonds, magenta dotted lines mark stacked or T-shaped Pi–Pi interactions, black dotted lines mark zinc chelation, and the brown dotted line indicates an amide–Pi-stacked interaction.

In order to gain a deeper understanding of the mechanisms of action of the new HDAC11 inhibitors, molecular docking was first used to investigate how the inhibitors bind to HDAC11 and which molecular interactions are formed. Since a crystal structure of HDAC11 does not yet exist, an AlphaFold structure optimized by Sippl et al. was used as a receptor [32]. In order to explain the experimental findings for isoenzyme selectivities, the most active substances were also docked into known X-ray crystal structures of HDAC1 (PDB-ID: 4BKX), HDAC4 (PDB-ID: 4CBY), HDAC6 (PDB-ID: 5EDU), and HDAC8 (PDB-ID: 3SFF). The most potent substance from cluster #1, hydroxamic acid 9HDA020C06, was the most active HDAC11 inhibitor (IC_50_ = 1.1 ± 0.4 µM), but at the same time inhibited HDAC1, 4, 6, and 8 in a similar concentration range (Table 2). This also applies to other active cluster #1 substances, which are structurally very similar. The experimental pan-inhibitory properties are in agreement with the high negative docking scores for all HDAC complexes examined with 9HDA020C06 (Table S3). Likewise, this compound binds similarly to all HDACs examined (Figure S4). In particular, the catalytic zinc ion in the binding pocket of all HDAC isozymes is chelated in the same way via the carbonyl oxygen of the hydroxamic acid. The para-substitution of benzene hydroxamic acid allows access of the hydroxamate warhead to the active site of all investigated HDAC crystal structures, especially HDAC6. The para-substituent points outwards and can interact with the outer edge of the binding funnel (Figure S4). The selective HDAC11 inhibitor CAPE emerged from cluster #2. Also in this cluster, strongly negative GBVI/WSA dG scores of < −9 are associated with micromolar IC_50_ values, while the non-active catechol-cinnamic acid (9LOP003E09) has a score of only −7.2, in agreement with the experimental values (Table S4). Among the 10,281 screened compounds, there were 88 substances containing the catechol group. Only six of them showed inhibitory effects (<70% residual HDAC11 activity). Therefore, the catechol group on its own appeared not the determining contribution to binding affinity. CAPE was then docked into the other HDAC isoenzymes as described above. Again, there was good agreement between the experimental activities in terms of IC_50_ values and the docking scores, confirming that the docking procedure yields reliable results and that CAPE is a selective HDAC11 inhibitor (Table S5 and Figure S5). Docking poses of CAPE in HDAC11 show that zinc-binding occurs through the carbonyl oxygen (Figure 6A). The binding of CAPE is further stabilized by a hydrogen bridge to the side chain of E94, Pi–Pi interactions with Y209 and H183, and an amide–Pi stacking interaction. The binding mode can also explain why the activity decreases significantly when the free acid is tested instead of the phenylethyl ester. The phenylethyl residue protrudes into the hydrophobic channel, which in the case of the HDAC11–SIS17 complex is filled by the alkyl chain of SIS17 [33]. Presumably, there are other hydrophobic interactions that were not visible in the docked complex structure with the AlphaFold model of HDAC11 used. The singleton hit compound SPC045H03 was shown to be a selective HDAC11 with good activity (Table 1 and Figure S8). SPC045H03 was tested as a racemate. In order to clarify which enantiomer would exert higher activity, we performed docking with both enantiomers using the AlphaFold model of HDAC11. It turned out that both enantiomers showed similar docking scores: −9.9 for the R enantiomer and −9.5 for the S enantiomer. Also, both enantiomers of SPC045H03 show similar zinc-binding through its pyridine nitrogen (Figure 6B and Figure S6). In addition, 9SPC045H03 forms a hydrogen bond to the carbonyl oxygen of G140 and multiple stacked or T-shaped Pi–Pi interactions with F153, Y209, and H143 (Figure 6B), explaining the single-digit micromolar activity against HDAC11. To understand the observed isoenzyme selectivity, 9SPC045H03 was also docked into the crystal structures of HDACs 1, 4, 6, and 8. The docking score for the complex between HDAC11 and the R-enantiomer of 9SPC045H03 was significantly better (−9.9) than the best score for other HDAC complexes (≥−8.8), in agreement with the experimental IC_50_ values (Table S6).

A superposition of the binding pose of CAPE and SIS17 shows that a phenyl group protrudes into the hydrophobic tunnel occupied by the alkyl group in the AlphaFold structure of the HDAC11–SIS17 complex (Figure 6A). This therefore offers the possibility of extending CAPE in this direction using medicinal chemistry. Since the binding pose of 9SPC045H03 is very unusual and the structure class is easily accessible medico-chemically by the Mannich reaction, a ligand-based optimization via the quantitative structure–activity relationship appears suitable for this lead structure.

## 3. Material and Methods

### 3.1. High-Throughput Screening

The screening was performed using the commercially available compound libraries LOPAC^®^1280 (Merck, Darmstadt, Germany), SPECS compound library (eMolecules, Zoetermeer, The Netherlands), and the in-house assembled HDA compound library. The compounds were dried in 96-well half-area plates. Residual enzyme activity was determined by adding 50 nM HDAC11 in an MAL buffer (50 mM Tris-HCl (pH8), 137 mM NaCl, 2.7 mM KCl, 1 mM MgCl_2_, 0.5% BSA) to the plates and incubating at 30 °C for 30 min. The final compound concentration in the assay was 10 µM. The enzyme activity assay was then performed as described below. The hit threshold was determined from the Gaussian distribution of residual enzyme activities in the presence of compounds, defined as the lower four-fold standard deviation from the mean representing non-active compounds.

### 3.2. Production and Purification of HDAC11

Codon optimized (BioCat GmbH, Heidelberg, Germany) HDAC11 (Uniprot: Q96DB2-1) was produced as an N-terminal His6-SUMO tagged fusion protein in *E. coli* BL21 (DE3) cells in LB media. Cells were grown to an OD600 of 0.5 (37 °C; 180 rpm) and then cooled at 16 °C for 30 min before being induced with 0.2 mM IPTG and grown at 16 °C overnight. Cells were harvested by centrifugation at 4000× *g* for 10 min at 4 °C. The pellet was resuspended in an IMAC A buffer (50 mM Tris-HCl, 150 mM KCl, 10 mM imidazole, pH 8), and a few crystals of lysozyme and 100 µg/mL DNAse I were added. The suspension was sonicated on ice for 20 min (2 s pulse/4 s pause) and then centrifuged at 18,000× *g* for 40 min at 4 °C. The lysate was further prepared as described by Rohman et al [32]. Briefly, 10 mM ATP was added to the lysate and incubated for 30 min at 4 °C. Denatured bacterial lysate was added and incubated for an additional 40 min at 4 °C and then centrifuged at 4000× *g* for 5 min. The lysate was applied to a Ni^2+^-IDA column and washed with an IMAC A buffer and ATP wash buffer (5 mM ATP, 50 mM Tris-HCL, 400 mM KCl, 20 mM MgCl_2_, pH 8). Protein was eluted with an IMAC B buffer (50 mM Tris-HCl, 150 mM KCl, 500 mM imidazole, pH 8) and concentrated, and the buffer was exchanged to an SEC buffer (50 mM Tris-HCl, 150 mM KCl, 5% glycerol, 1 mM TCEP, pH 8) by ultrafiltration. Protein concentrations were determined by measuring absorbance at 280 nm using the extinction coefficients of the proteins and stored at −80 °C.

### 3.3. Enzyme Activity Assay

HDAC11 activity was determined using a fluorimetric assay as previously described [34]. HDAC11 (50 nM) was incubated with a serial dilution of the compounds for the indicated times at 30 °C in an MAL buffer. The enzyme reaction was initiated by the addition of 80 μM of the substrate Boc-Lys(TFA)-AMC (Bachem, Bubendorf, Switzerland). After 60 min, the reaction was stopped and developed by adding 20 µM FT894 (Merck, Darmstadt, Germany) and 0.42 mg/mL trypsin (AppliChem, Darmstadt, Germany). Measurements were performed using a fluorescence microplate reader (PHERAstar FS, BMG LABTECH, Ortenberg, Germany) with fluorescence excitation at 360 nm and emission at 460 nm. IC50 values were calculated using GraphPad Prism 9 by generating dose-response curves and fitting them to a 4-parameter model.

### 3.4. Time-Dependent IC50 Values

A one-step enzyme assay was used to determine time-dependent IC_50_ values, as previously described [35]. After the indicated pre-incubation times, 50 nM HDAC11 was added to a serial dilution of a master mix consisting of an 80 µM substrate (Boc-Lys(TFA)-AMC) and 0.2 mg/mL trypsin. Progression curves were then measured immediately using a fluorescence microplate reader (PHERAstar FS, BMG LABTECH), with excitation at 360 nm and emission at 460 nm. HDAC11 activity was determined from the slope of the progression curve.

### 3.5. Rapid Dilution

The inhibitors were evaluated for reversible inhibition in an enzyme activity assay. Compounds at 10× the IC_50_ value were incubated with HDAC11 at 100× the normal concentration. After equilibration for 1 h at 30 °C, this solution is diluted 100-fold directly into 80 μM of the substrate Boc-Lys(TFA)-AMC. After a further hour of incubation, the reaction was stopped as indicated in the enzyme activity assay section. As a control, compounds were tested at 100× the IC_50_ and at 10× the normal assay concentration of HDAC11. After ten-fold dilution, this control should still inhibit the enzyme and therefore show that 10× the IC_50_ value can almost completely inhibit HDAC11.

### 3.6. Molecular Docking

Modeling, preparation, and visualization of structural data, as well as molecular docking, were performed using MOE 2024.0601 software (Chemical Computing Group ULC, Montreal, QC, Canada). Since there is no experimental 3D structure of HDAC11, the AlphaFold structure of the complex of HDAC11 with SIS17 optimized by Sippl et al. was used here [33]. To map the isoenzyme selectivity, the following X-ray crystal structures were used as the receptor: HDAC1 (PDB ID: 4BKX), HDAC4 (PDB ID: 4CBY), HDAC6 (PDB ID: 5EDU), and HDAC8 (PDB ID: 3SFF). All receptor structures were subjected to the Quickprep procedure of MOE 2024, including 3D protonation for subsequent docking. The partial charges of all protein and ligand atoms were calculated using the implemented Amber EHT force field. The docking site was defined by the ligand within the binding pocket of the respective protein structure. Molecular docking was performed by choosing the triangle matcher for the placement of the ligand in the binding site and ranked with the London dG scoring function. The best 50 poses were passed onto refinement and energy minimization in the pocket using the induced fit method, and the 10 best poses were rescored using the GBVI/WSA dG scoring function.

## 4. Conclusions

HDAC11 has been identified as a very promising target for cancer, inflammatory diseases, and metabolic disorders. To date, there is no experimental 3D structure of the enzyme that could be utilized for rational drug design. Therefore, we conducted an unbiased experimental screening campaign on a structurally diverse compound library. The hit rate was remarkably low at 0.8%. We have identified two novel non-hydroxamate HDAC11 inhibitors that exhibit IC_50_ values of (1.5 ± 0.4) and (2.3 ± 0.5) µM, remarkable isozyme selectivity, and rapid reversible binding to HDAC11. In addition, the compounds possess favorable drug-like physicochemical properties. Molecular docking with an optimized AlphaFold structure revealed that the new inhibitors coordinate the catalytic zinc ion in the active site via carbonyl oxygen (CAPE) or the nitrogen atom in a pyridine residue (9SPC045H03). Structurally, the two compounds are entirely distinct from the few currently known HDAC11 inhibitors and represent novel scaffolds for the development of selective HDAC11 inhibitors that avoid the metabolically problematic and potentially mutagenic hydroxamic acid warhead.

## Figures and Tables

**Table 1:** IC_50_ values of hit compounds against HDAC11. IC_50_ values are given as the means ± standard deviation (n = 3).

Cluster #	Structure	ID	IC_50_ (µM)
1	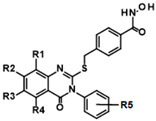		
	R1:H,R2:H,R3H,R4Me,R5:H	9HDA020C06	1.1 ± 0.4
	R1:H,R2:H,R3H,R4:Me,R5:p-Cl	9HDA020C09	1.9 ± 0.4
	R1:H,R2:H,R3H,R4:Me,R5:o-F	9HDA020C07	1.5 ± 0.3
	R1:H,R2:H,R3H,R4:Me,R5:p-Et	9HDA020B06	1.6 ± 0.2
	R1:H,R2:H,R3H,R4:H,R5:m-Me	9HDA020B09	1.6 ± 0.3
	R1:H,R2:H,R3H,R4:H,R5:p-Me	9HDA020B05	2.0 ± 0.2
	R1:H,R2:Cl,R3H,R4:H,R5:o-F	9HDA020C04	2.0 ± 0.5
	R1:H,R2:H,R3H,R4:H,R5:o-Me,p-Me	9HDA020B08	2.0 ± 0.2
	R1:H,R2:H,R3H,R4:H,R5:m-Cl	9HDA020B10	2.0 ± 0.3
	R1:H,R2:H,R3H,R4:H,R5:H	9HDA020B04	2.1 ± 0.2
	R1:H,R2:H,R3H,R4:H,R5:p-Br	9HDA020B07	2.6 ± 0.4
	R1:H,R2:H,R3H,R4:Me,R5:o-Me	9HDA020C08	3.5 ± 0.8
	R1:H,R2:H,R3H,R4:H,R5:o-F	9HDA020B11	4.1 ± 0.5
2	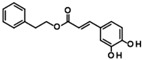	9LOP004C10 (CAPE)	1.5 ± 0.4
	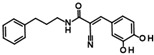	9LOP015D09	4.3 ± 3
	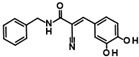	9LOP015E07	14 ± 3
5	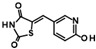	9HDA018E08	19 ± 2
6	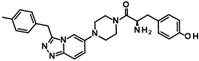	9HDA027C02	>50
Singleton	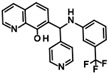	9SPC045H03	2.3 ± 0.5
Singleton	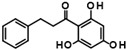	9SPC036F04	>50
Reference	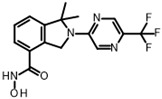	FT895	0.003 ± 0.001

**Table 2:** Isoenzyme selectivity of selected HDAC11 inhibitors. IC_50_ values are given as the means ± standard deviation (n = 3).

		IC_50_ (µM)				
Cluster #	ID	HDAC1	HDAC4	HDAC6	HDAC8	HDAC11
1	9HDA020C06	1.4 ± 0.4	0.4 ± 0.2	0.2 ± 0.1	2.7 ± 0.6	1.1 ± 0.4
	9HDA020C09	2.3 ± 0.5	0.9 ± 0.4	0.9 ± 0.3	6 ± 1	1.9 ± 0.4
2	CAPE	22 ± 5	68 ± 30	>70	7 ± 2	1.5 ± 0.4
Singleton	9SPC045H03	>70	>70	67	13 ± 5	2.3 ± 0.5
Reference	FT895	>10 *	>10 *	>10 *	5.6 *	0.003 ± 0.001

* [4]. # is the number of the cluster.

## Data Availability

Data is contained within the article or Supplementary Materials.

## Supplementary Materials

The following supporting information can be downloaded at: https://www.mdpi.com/article/10.3390/ijms26135950/s1.
