# Supplementary material for: Discovery and Characterization of Novel Non-Hydroxamate HDAC11 Inhibitors
Source: Int J Mol Sci. 2025 Jun 20;26(13):5950. doi: 10.3390/ijms26135950 (PMC12249833; doi:10.3390/ijms26135950)
Supplement: Supplementary file 1 [file ijms-26-05950-s001.zip › ijms-3642263-supplementary.pdf]

Supporting Information

Discovery and characterization of novel non-hydroxamate HDAC11 inhibitors

Aleksandra Kopranovic[a,b], Franz-Josef Meyer-Almes*[a,b]

[a]*Department of Chemical Engingeering and Biotechnologiy, Darmstadt University of Applied Sciences, Haardtring 100, 64295 Darmstadt, Germany.*
[b] *European University of Technology, European Union*

Content:

- Table S1: HDAC11 activities of structurally similar substances to CAPE. Enzyme activities are given in % and IC_{50}-values in µM.
- Table S2: HDAC11 activities of structurally similar substances to thiazolidinedione 9HDA018E08. Enzyme activities are given in % and IC_{50}-values in µM.
- Table S3: Docking scores for cluster #1 hit 9HDA020C06 in panel of HDAC-isozyme structures.
- Table S4: Docking scores for cluster #2 compounds in HDAC11.
- Table S5: Docking scores for cluster #2 hit CAPE in panel of HDAC-isozyme structures.
- Table S6: Docking scores for 9SPC045H03 enantiomers (R and S).
- Figure S1: Confirmation of potent singleton hits with residual HDAC11 activity < 50% in the presence of 35 µM compound.
- Figure S2: Calculated ADME parameters of best hits and reference compounds.
- Figure S3: Dose-response curves of 9SPC045H03 and CAPE against a panel of HDAC isozymes.
- Figure S4: Superposed docking poses of cluster #1 hit 9HDA020C06 (orange) in complex with HDACs 1 (PDB-ID: 4BKX), 4 (PDB-ID: 4CBY), 6 (PDB-ID: 5EDU), 8 (PDB-ID: 3SFF) and 11 (AlphaFold structure)
- Figure S5: Correlation of docking scores and experimental IC_{50}-values.
- Figure S6: Overlay of similar binding poses of R- (green) and S- (dark pink) enantiomer of 9SPC045H03.
- Figure S7: Timedependent dose-response curves of indicated compounds.
- Figure S8: Dose-response curves of potent hit-compounds against HDAC11
- Figure S9: Rapid dilution experiments to demonstrate the reversibility of binding of the indicated compounds to HDAC11.
- Figure S10: Cytotoxicity testing of indicated compound against HEK293 cell.
- Figure S11: Control experiment to demonstrate that the inhibitory effect of CAPE and 9SPC045H03 in the enzyme activity assay is not due to compound-substrate interference.
- Figure S12: Correlation between published and experimental IC_{50}-values.

Table S1: HDAC11 activities of structurally similar substances to CAPE. Enzyme activities are given in % and IC_{50}-values in µM.

Structure	Subst ID	Root Name	HDAC11 Enz Act	HDAC11 IC50
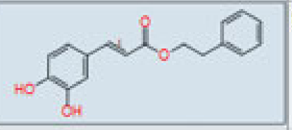	9LOP004C10	CAPE	46	1.2
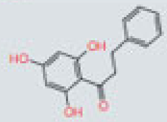	9SPC036F04	3-phenyl-1-(2,4,6-trihydroxyphenyl)-1-propanone	46	62
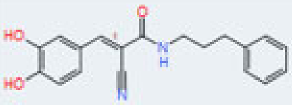	9LOP015D09	Tyrphostin B46 AG555	49	4,3
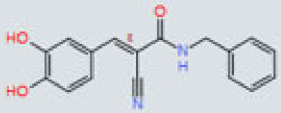	9LOP015E07	Tyrphostin B42, AG490	65	14
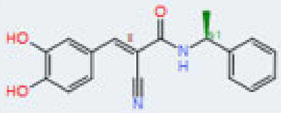	9LOP015H09		74	
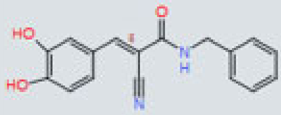	9HDA005E09	AG 490	76.74861511	
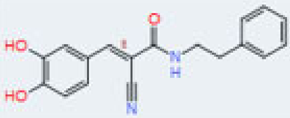	9LOP015E09		77	
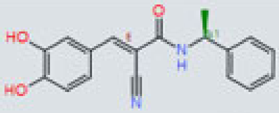	9LOP015F08		79	
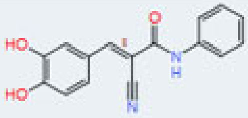	9LOP015C08		79	
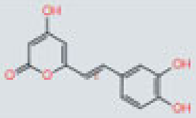	9LOP008H05		82	
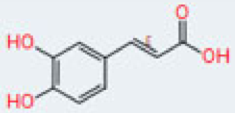	9LOP003E09		90	
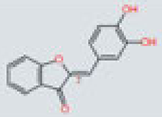	9SPC108E11	2-(3,4-dihydroxybenzylidene)-1-benzofuran-3(2H)-one	91	
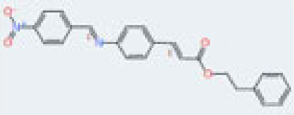	9SPC056H05	2-phenylethyl 3-[4-({4-nitrobenzylidene}amino)phenyl]acrylate	97	

Table S2: HDAC11 activities of structurally similar substances to thiazolidinedione 9HDA018E08. Enzyme activities are given in % and IC_{50}-values in µM.

Structure	Subst ID	Root Name	HDAC11 Enz Act	HDAC11 IC50
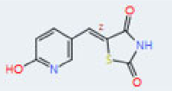	9HDA018E08	01b	28.55369796	19
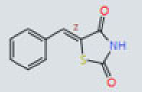	9HDA018F03	01h	71.94924281	
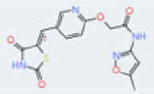	9HDA013G04	5t	81.63149495	
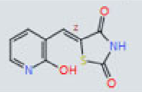	9HDA018E09	01c	81.68573149	
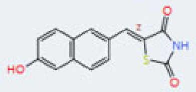	9HDA018E07	01a	82.08586116	
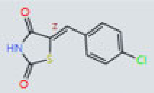	9SPC030A07	5-(4-chlorobenzylidene)-1,3-thiazolidine-2,4-dione	86	
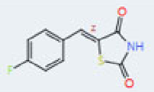	9HDA018F04	01i	86.30204233	
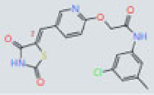	9HDA011F08	5u	87.26731366	
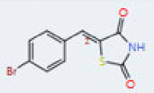	9HDA018G02	01r	87.41536609	
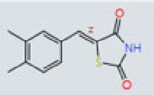	9HDA018F05	01j	87.85624971	
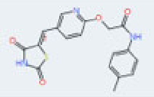	9HDA011E10	5c	88.9284395	
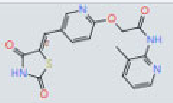	9HDA013B04	5n	90.01359579	
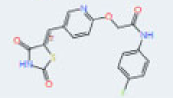	9HDA013G11	5b	90.38954897	
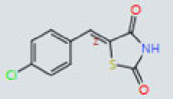	9HDA018F11	01p	95.7143518786	
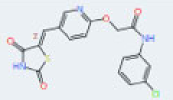	9HDA011E08	5f	91.07984374	
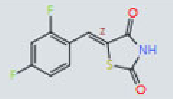	9HDA018F10	01o	93.26541009	
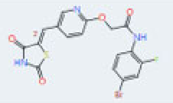	9HDA011F02	5r	93.57377123	
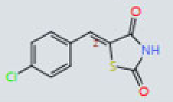	9HDA018F02	01g	101.610707286	
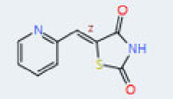	9HDA018E10	01d	94.98819062	
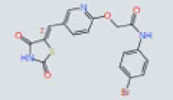	9HDA011F07	5o	95.01774181	
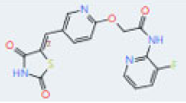	9HDA013E06	5s	95.46137022	

Table S3: Docking scores for cluster #1 hit 9HDA020C06 in panel of HDAC-isozyme structures.

**Receptor**	**Score (GBVI/WSA dG)**	**Res. HDAC activity (%)**	**IC_{50} (µM)**	**Zn^{2+}-binding**
HDAC11	-9.0	58	1.1 ± 0.4	Carbonyl-O-Zn^{2+}
HDAC1 (4BKX)	-8.7	-	1.4	Carbonyl-O-Zn^{2+}
HDAC4 (4CBY)	-8.6	-	0.4	Carbonyl-O-Zn^{2+}
HDAC6 (5EDU)	-8.8	-	0.22	Carbonyl-O-Zn^{2+}
HDAC8 (3SFF)	-8.7	-	2.7	Carbonyl-O-Zn^{2+}

Table S4: Docking scores for cluster #2 compounds in HDAC11:

Cpd	Score (GBVI/WSA dG)	Res. HDAC activity (%)	IC_{50} (µM)	Zn^{2+}-binding
9LOP004C10 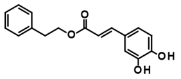	-9.1	46	1.5 ± 0.4	Carbonyl-O-Zn^{2+}
9LOP015D09 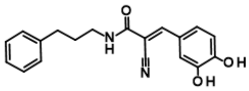	-9.6	49	4.3 ± 1.3	Carbonyl-O-Zn^{2+}
9LOP015E07 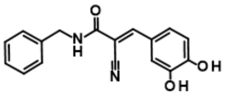	-9.3	65	14 ± 3	Carbonyl-O-Zn^{2+}
9LPOP003E09 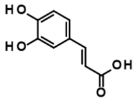 Inactive Control	-7.2	90	>100	Carbonyl-O-Zn^{2+}

Table S5: Docking scores for cluster #2 hit CAPE in panel of HDAC-isozyme structures.

Receptor	Score (GBVI/WSA dG)	Res. HDAC activity (%)	IC_{50} (µM)	Zn^{2+}-binding
HDAC11	-9.1	46	1.5 ± 0.4	Carbonyl-O-Zn^{2+}
HDAC1 (4BKX)	-9.2	-	22	Carbonyl-O-Zn^{2+}
HDAC4 (4CBY)	-7.6	-	43	Carbonyl-O-Zn^{2+}
HDAC6 (5EDU)	-7.7	-	70	Carbonyl-O-Zn^{2+}
HDAC8 (3SFF)	-8.4	-	5.9	Carbonyl-O-Zn^{2+}

Table S6: Docking scores for 9SPC045H03 enantiomers (R and S) in agreement with selective HDAC11 inhibition

**Receptor**	**Cpd**	**Score (GBVI/WSA dG)**	**Res. HDAC activity (%)**	**IC_{50} (µM)**	**Zn^{2+}-binding**
HDAC11	9SPC045H03_R	-9.9	47	2.3 ± 0.5	Pyridine-Nitrogen
HDAC11	9SPC045H03_S	-9.5	47	2.3 ± 0.5	Pyridine-Nitrogen
HDAC1 (4BKX)	9SPC045H03_R	-8.5	-	>70	Quinoline-OH
HDAC4 (4CBY)	9SPC045H03_R	-8.8	-	>70	Quinoline-N
HDAC6 (5EDU)	9SPC045H03_R	-7.7	-	67	none
HDAC8 (3SFF)	9SPC045H03_R	-8.0	-	7.8 ± 0.1	Quinoline-N

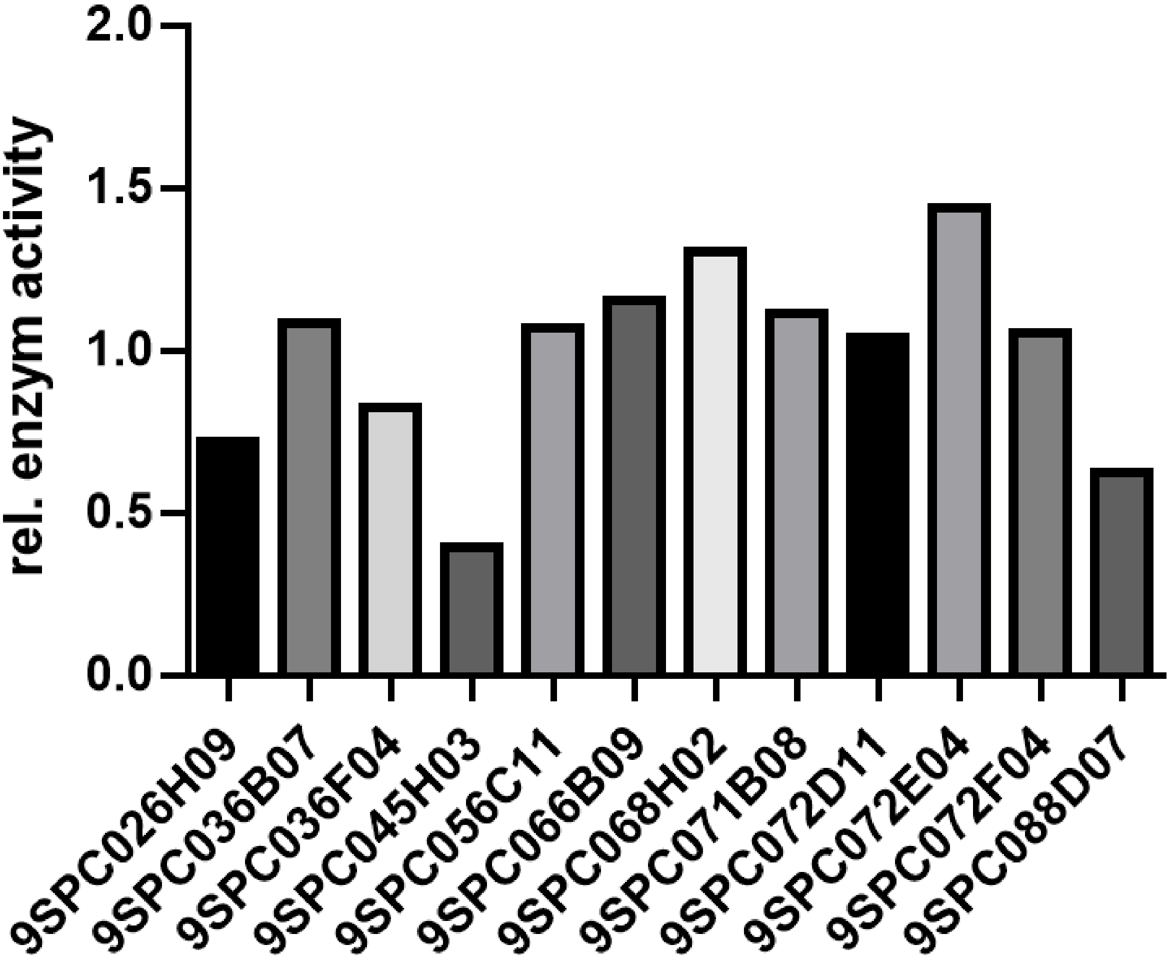

Figure S1: Confirmation of potent singleton hits with residual HDAC11 activity < 50% in the presence of 35 µM compound.

Molecule 1

FT895

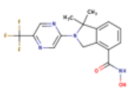

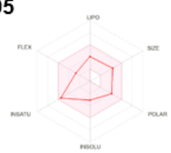

SMILES ONC(=O)c1cccc2c1CN(C2(C)C)c1cnc(cn1)C(F)(F)F

Physicochemical Properties	
Formula	C16H15F3N4O2
Molecular weight	352.31 g/mol
Num. heavy atoms	25
Num. arom. heavy atoms	12
Fraction Csp3	0.31
Num. rotatable bonds	4
Num. H-bond acceptors	7
Num. H-bond donors	2
Molar Refractivity	85.12
TPSA	78.35 Å²

Lipophilicity	
Log $P_{o/w}$ (iLOGP)	0.58
Log $P_{o/w}$ (XLOGP3)	1.60
Log $P_{o/w}$ (WLOGP)	3.38
Log $P_{o/w}$ (MLOGP)	1.86
Log $P_{o/w}$ (SILICOS-IT)	2.27
Consensus Log $P_{o/w}$	1.94

Water Solubility	
Log *S* (ESOL)	-3.12
Solubility	2.65e-01 mg/ml ; 7.52e-04 mol/l
Class	Soluble
Log *S* (Ali)	-2.86
Solubility	4.90e-01 mg/ml ; 1.39e-03 mol/l
Class	Soluble
Log *S* (SILICOS-IT)	-5.09
Solubility	2.86e-03 mg/ml ; 8.11e-06 mol/l
Class	Moderately soluble

Pharmacokinetics	
GI absorption	High
BBB permeant	Yes
P-gp substrate	Yes
CYP1A2 inhibitor	No
CYP2C19 inhibitor	No
CYP2C9 inhibitor	No
CYP2D6 inhibitor	No
CYP3A4 inhibitor	No
Log K_p (skin permeation)	-7.31 cm/s

Druglikeness	
Lipinski	Yes; 0 violation
Ghose	Yes
Veber	Yes
Egan	Yes
Muegge	Yes
Bioavailability Score	0.55

Medicinal Chemistry	
PAINS	0 alert
Brenk	2 alerts: hydroxamic_acid, oxygen-nitrogen_single_bond
Leadlikeness	No; 1 violation: MW>350
Synthetic accessibility	2.82

Molecule 2

TD045

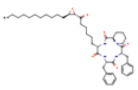

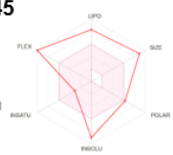

SMILES CCCCCCCCCCC[C@H]1O[C@@H]1C(=O)CCCCC[C@@H]1NC(=O)[C@H]2CCCCN2C(=O)[C@@H](NC(=O)[C@@H](NC1=O)Cc1ccccc1)Cc1ccccc1

Physicochemical Properties	
Formula	C45H64N4O6
Molecular weight	757.01 g/mol
Num. heavy atoms	55
Num. arom. heavy atoms	12
Fraction Csp3	0.62
Num. rotatable bonds	21
Num. H-bond acceptors	6
Num. H-bond donors	3
Molar Refractivity	232.42
TPSA	137.21 Å²

Lipophilicity	
Log $P_{o/w}$ (iLOGP)	5.85
Log $P_{o/w}$ (XLOGP3)	9.07
Log $P_{o/w}$ (WLOGP)	5.01
Log $P_{o/w}$ (MLOGP)	2.91
Log $P_{o/w}$ (SILICOS-IT)	8.61
Consensus Log $P_{o/w}$	6.29

Water Solubility	
Log *S* (ESOL)	-9.02
Solubility	7.18e-07 mg/ml ; 9.48e-10 mol/l
Class	Poorly soluble
Log *S* (Ali)	-11.84
Solubility	1.08e-09 mg/ml ; 1.43e-12 mol/l
Class	Insoluble
Log *S* (SILICOS-IT)	-12.89
Solubility	9.77e-11 mg/ml ; 1.29e-13 mol/l
Class	Insoluble

Pharmacokinetics	
GI absorption	Low
BBB permeant	No
P-gp substrate	Yes
CYP1A2 inhibitor	No
CYP2C19 inhibitor	No
CYP2C9 inhibitor	No
CYP2D6 inhibitor	No
CYP3A4 inhibitor	Yes
Log K_p (skin permeation)	-4.48 cm/s

Druglikeness	
Lipinski	Yes; 1 violation: MW>500
Ghose	No; 3 violations: MW>480, MR>130, #atoms>70
Veber	No; 1 violation: Rotors>10
Egan	No; 1 violation: TPSA>131.6
Muegge	No; 3 violations: MW>600, XLOGP3>5, Rotors>15
Bioavailability Score	0.55

Medicinal Chemistry	
PAINS	0 alert
Brenk	1 alert: Three-membered_heterocycle
Leadlikeness	No; 3 violations: MW>350, Rotors>7, XLOGP3>3.5
Synthetic accessibility	7.14

Molecule 3

14-NC6-OH

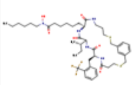

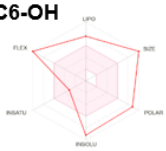

SMILES CCCCCCN(C(=O)CCCCC[C@@H]1NC(=O)C(NC(=O)[C@@H](NC(=O)CCSCc2cc(CSCCCNC1=O)ccc2)Cc1ccccc1C(F)(F)F)[C@H](CC)C)O

Physicochemical Properties	
Formula	C44H64F3N5O6S2
Molecular weight	880.13 g/mol
Num. heavy atoms	60
Num. arom. heavy atoms	12
Fraction Csp3	0.61
Num. rotatable bonds	17
Num. H-bond acceptors	9
Num. H-bond donors	5
Molar Refractivity	249.83
TPSA	207.54 Å²

Lipophilicity	
Log $P_{o/w}$ (iLOGP)	6.27
Log $P_{o/w}$ (XLOGP3)	7.75
Log $P_{o/w}$ (WLOGP)	6.90
Log $P_{o/w}$ (MLOGP)	3.71
Log $P_{o/w}$ (SILICOS-IT)	8.00
Consensus Log $P_{o/w}$	6.52

Water Solubility	
Log *S* (ESOL)	-9.21
Solubility	5.49e-07 mg/ml ; 6.23e-10 mol/l
Class	Poorly soluble
Log *S* (Ali)	-11.95
Solubility	9.84e-10 mg/ml ; 1.12e-12 mol/l
Class	Insoluble
Log *S* (SILICOS-IT)	-13.04
Solubility	8.08e-11 mg/ml ; 9.18e-14 mol/l
Class	Insoluble

Pharmacokinetics	
GI absorption	Low
BBB permeant	No
P-gp substrate	Yes
CYP1A2 inhibitor	No
CYP2C19 inhibitor	No
CYP2C9 inhibitor	No
CYP2D6 inhibitor	No
CYP3A4 inhibitor	Yes
Log K_p (skin permeation)	-6.17 cm/s

Druglikeness	
Lipinski	No; 2 violations: MW>500, NorO>10
Ghose	No; 4 violations: MW>480, WLOGP>5.6, MR>130, #atoms>70
Veber	No; 2 violations: Rotors>10, TPSA>140
Egan	No; 2 violations: WLOGP>5.88, TPSA>131.6
Muegge	No; 4 violations: MW>600, XLOGP3>5, TPSA>150, Rotors>15
Bioavailability Score	0.17

Medicinal Chemistry	
PAINS	0 alert
Brenk	2 alerts: hydroxamic_acid, oxygen-nitrogen_single_bond
Leadlikeness	No; 3 violations: MW>350, Rotors>7, XLOGP3>3.5
Synthetic accessibility	8.43

Molecule 4

Trapoxin A

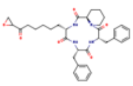

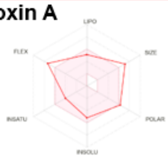

SMILES O=C1N[C@@H](Cc2ccccc2)C(=O)N[C@@H](Cc2ccccc2)C(=O)N2[C@@H](C(=O)N[C@H]1CCCCCC(=O)C1CO1)CCCC2

Physicochemical Properties	
Formula	C34H42N4O6
Molecular weight	602.72 g/mol
Num. heavy atoms	44
Num. arom. heavy atoms	12
Fraction Csp3	0.50
Num. rotatable bonds	11
Num. H-bond acceptors	6
Num. H-bond donors	3
Molar Refractivity	179.55
TPSA	137.21 Å²

Lipophilicity	
Log $P_{o/w}$ (iLOGP)	2.90
Log $P_{o/w}$ (XLOGP3)	3.42
Log $P_{o/w}$ (WLOGP)	0.72
Log $P_{o/w}$ (MLOGP)	1.02
Log $P_{o/w}$ (SILICOS-IT)	4.31
Consensus Log $P_{o/w}$	2.47

Water Solubility	
Log *S* (ESOL)	-5.21
Solubility	3.74e-03 mg/ml ; 6.20e-06 mol/l
Class	Moderately soluble
Log *S* (Ali)	-5.98
Solubility	6.29e-04 mg/ml ; 1.04e-06 mol/l
Class	Moderately soluble
Log *S* (SILICOS-IT)	-8.91
Solubility	7.42e-07 mg/ml ; 1.23e-09 mol/l
Class	Poorly soluble

Pharmacokinetics	
GI absorption	High
BBB permeant	No
P-gp substrate	Yes
CYP1A2 inhibitor	No
CYP2C19 inhibitor	No
CYP2C9 inhibitor	No
CYP2D6 inhibitor	No
CYP3A4 inhibitor	Yes
Log K_p (skin permeation)	-7.55 cm/s

Druglikeness	
Lipinski	Yes; 1 violation: MW>500
Ghose	No; 3 violations: MW>480, MR>130, #atoms>70
Veber	No; 1 violation: Rotors>10
Egan	No; 1 violation: TPSA>131.6
Muegge	No; 1 violation: MW>600
Bioavailability Score	0.55

Medicinal Chemistry	
PAINS	0 alert
Brenk	1 alert: Three-membered_heterocycle
Leadlikeness	No; 2 violations: MW>350, Rotors>7
Synthetic accessibility	5.53

Molecule 5

PB94

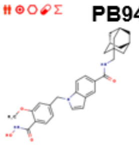

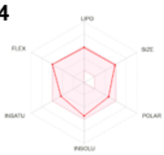

SMILES ONC(=O)c1ccc(cc1OC)Cn1ccc2c1ccc(c2)C(=O)NCC12C[C@@H]3C[C@H](C2)C[C@H](C1)C3

Physicochemical Properties	
Formula	C29H33N3O4
Molecular weight	487.59 g/mol
Num. heavy atoms	36
Num. arom. heavy atoms	15
Fraction Csp3	0.45
Num. rotatable bonds	9
Num. H-bond acceptors	4
Num. H-bond donors	3
Molar Refractivity	137.56
TPSA	92.59 Å²

Lipophilicity	
Log $P_{o/w}$ (iLOGP)	2.97
Log $P_{o/w}$ (XLOGP3)	4.54
Log $P_{o/w}$ (WLOGP)	4.76
Log $P_{o/w}$ (MLOGP)	3.56
Log $P_{o/w}$ (SILICOS-IT)	3.83
Consensus Log $P_{o/w}$	3.93

Water Solubility	
Log S (ESOL)	-5.44
Solubility	1.78e-03 mg/ml ; 3.65e-06 mol/l
Class	Moderately soluble
Log S (Ali)	-6.21
Solubility	3.03e-04 mg/ml ; 6.21e-07 mol/l
Class	Poorly soluble
Log S (SILICOS-IT)	-7.24
Solubility	2.81e-05 mg/ml ; 5.75e-08 mol/l
Class	Poorly soluble

Pharmacokinetics	
GI absorption	High
BBB permeant	No
P-gp substrate	Yes
CYP1A2 inhibitor	Yes
CYP2C19 inhibitor	No
CYP2C9 inhibitor	Yes
CYP2D6 inhibitor	Yes
CYP3A4 inhibitor	Yes
Log K_p (skin permeation)	-6.05 cm/s

Druglikeness	
Lipinski	Yes; 0 violation
Ghose	No; 2 violations: MW>480, MR>130
Veber	Yes
Egan	Yes
Muegge	Yes
Bioavailability Score	0.55

Medicinal Chemistry	
PAINS	0 alert
Brenk	2 alerts: hydroxamic_acid, oxygen-nitrogen_single_bond
Leadlikeness	No; 3 violations: MW>350, Rotors>7, XLOGP3>3.5
Synthetic accessibility	5.43

Molecule 6

D21

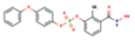

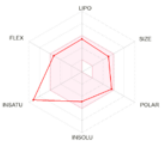

SMILES ONC(=O)c1cccc(c1C)OS(=O)(=O)Oc1ccc(cc1)Oc1ccccc1

Physicochemical Properties	
Formula	C20H17NO7S
Molecular weight	415.42 g/mol
Num. heavy atoms	29
Num. arom. heavy atoms	18
Fraction Csp3	0.05
Num. rotatable bonds	8
Num. H-bond acceptors	7
Num. H-bond donors	2
Molar Refractivity	103.50
TPSA	119.54 Å²

Lipophilicity	
Log $P_{o/w}$ (iLOGP)	3.16
Log $P_{o/w}$ (XLOGP3)	4.00
Log $P_{o/w}$ (WLOGP)	4.69
Log $P_{o/w}$ (MLOGP)	3.04
Log $P_{o/w}$ (SILICOS-IT)	1.67
Consensus Log $P_{o/w}$	3.31

Water Solubility	
Log S (ESOL)	-4.87
Solubility	5.64e-03 mg/ml ; 1.36e-05 mol/l
Class	Moderately soluble
Log S (Ali)	-6.21
Solubility	2.55e-04 mg/ml ; 6.13e-07 mol/l
Class	Poorly soluble
Log S (SILICOS-IT)	-6.30
Solubility	2.09e-04 mg/ml ; 5.02e-07 mol/l
Class	Poorly soluble

Pharmacokinetics	
GI absorption	Low
BBB permeant	No
P-gp substrate	No
CYP1A2 inhibitor	No
CYP2C19 inhibitor	Yes
CYP2C9 inhibitor	Yes
CYP2D6 inhibitor	No
CYP3A4 inhibitor	No
Log K_p (skin permeation)	-5.99 cm/s

Druglikeness	
Lipinski	Yes; 0 violation
Ghose	Yes
Veber	Yes
Egan	Yes
Muegge	Yes
Bioavailability Score	0.55

Medicinal Chemistry	
PAINS	0 alert
Brenk	2 alerts: hydroxamic_acid, oxygen-nitrogen_single_bond
Leadlikeness	No; 3 violations: MW>350, Rotors>7, XLOGP3>3.5
Synthetic accessibility	3.11

Molecule 7

Cpd 5a

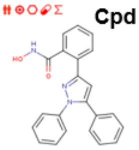

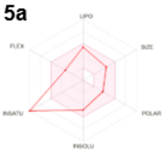

SMILES ONC(=O)c1ccccc1c1cc(n(n1)c1ccccc1)c1ccccc1

Physicochemical Properties	
Formula	C22H17N3O2
Molecular weight	355.39 g/mol
Num. heavy atoms	27
Num. arom. heavy atoms	23
Fraction Csp3	0.00
Num. rotatable bonds	5
Num. H-bond acceptors	3
Num. H-bond donors	2
Molar Refractivity	103.35
TPSA	67.15 Å²

Lipophilicity	
Log $P_{o/w}$ (iLOGP)	2.56
Log $P_{o/w}$ (XLOGP3)	4.00
Log $P_{o/w}$ (WLOGP)	4.33
Log $P_{o/w}$ (MLOGP)	3.43
Log $P_{o/w}$ (SILICOS-IT)	3.17
Consensus Log $P_{o/w}$	3.50

Water Solubility	
Log S (ESOL)	-4.86
Solubility	4.86e-03 mg/ml ; 1.37e-05 mol/l
Class	Moderately soluble
Log S (Ali)	-5.11
Solubility	2.75e-03 mg/ml ; 7.72e-06 mol/l
Class	Moderately soluble
Log S (SILICOS-IT)	-7.34
Solubility	1.61e-05 mg/ml ; 4.54e-08 mol/l
Class	Poorly soluble

Pharmacokinetics	
GI absorption	High
BBB permeant	Yes
P-gp substrate	No
CYP1A2 inhibitor	Yes
CYP2C19 inhibitor	Yes
CYP2C9 inhibitor	Yes
CYP2D6 inhibitor	Yes
CYP3A4 inhibitor	No
Log K_p (skin permeation)	-5.63 cm/s

Druglikeness	
Lipinski	Yes; 0 violation
Ghose	Yes
Veber	Yes
Egan	Yes
Muegge	Yes
Bioavailability Score	0.55

Medicinal Chemistry	
PAINS	0 alert
Brenk	2 alerts: hydroxamic_acid, oxygen-nitrogen_single_bond
Leadlikeness	No; 2 violations: MW>350, XLOGP3>3.5
Synthetic accessibility	2.96

Molecule 8

Quisinostat

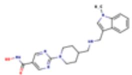

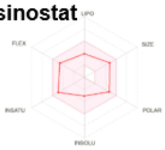

SMILES ONC(=O)c1cnc(nc1)N1CCC(CC1)CNCc1cn(c2c1cccc2)C

Physicochemical Properties	
Formula	C21H26N6O2
Molecular weight	394.47 g/mol
Num. heavy atoms	29
Num. arom. heavy atoms	15
Fraction Csp3	0.38
Num. rotatable bonds	7
Num. H-bond acceptors	5
Num. H-bond donors	3
Molar Refractivity	114.06
TPSA	95.31 Å²

Lipophilicity	
Log $P_{o/w}$ (iLOGP)	2.40
Log $P_{o/w}$ (XLOGP3)	1.33
Log $P_{o/w}$ (WLOGP)	1.56
Log $P_{o/w}$ (MLOGP)	1.33
Log $P_{o/w}$ (SILICOS-IT)	1.14
Consensus Log $P_{o/w}$	1.55

Water Solubility	
Log S (ESOL)	-3.04
Solubility	3.56e-01 mg/ml ; 9.03e-04 mol/l
Class	Soluble
Log S (Ali)	-2.93
Solubility	4.60e-01 mg/ml ; 1.17e-03 mol/l
Class	Soluble
Log S (SILICOS-IT)	-5.33
Solubility	1.87e-03 mg/ml ; 4.73e-06 mol/l
Class	Moderately soluble

Pharmacokinetics	
GI absorption	High
BBB permeant	No
P-gp substrate	Yes
CYP1A2 inhibitor	No
CYP2C19 inhibitor	No
CYP2C9 inhibitor	No
CYP2D6 inhibitor	Yes
CYP3A4 inhibitor	No
Log K_p (skin permeation)	-7.76 cm/s

Druglikeness	
Lipinski	Yes; 0 violation
Ghose	Yes
Veber	Yes
Egan	Yes
Muegge	Yes
Bioavailability Score	0.55

Medicinal Chemistry	
PAINS	0 alert
Brenk	2 alerts: hydroxamic_acid, oxygen-nitrogen_single_bond
Leadlikeness	No; 1 violation: MW>350
Synthetic accessibility	2.92

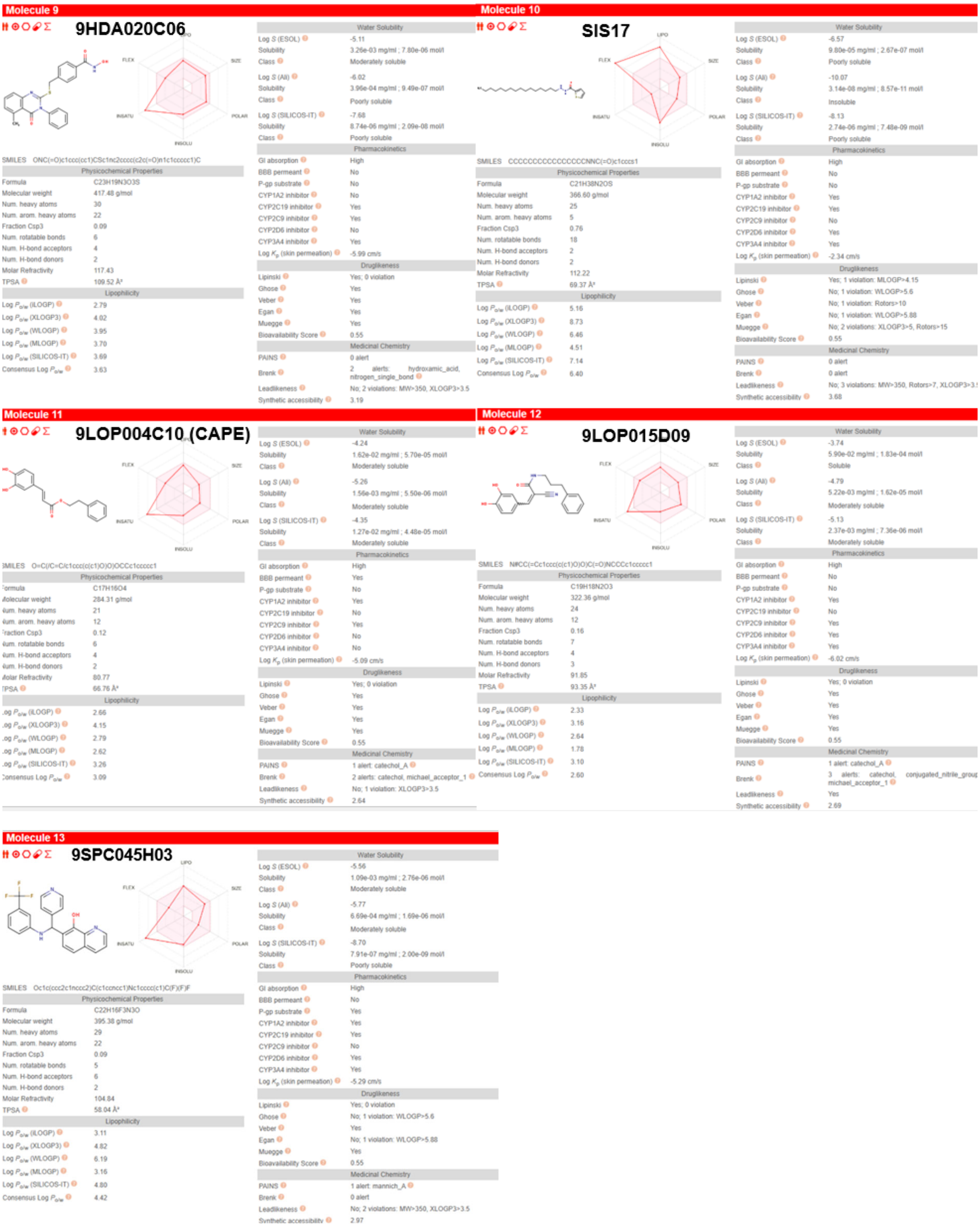

Figure S2: Calculated ADME parameters of best hits and reference compounds.

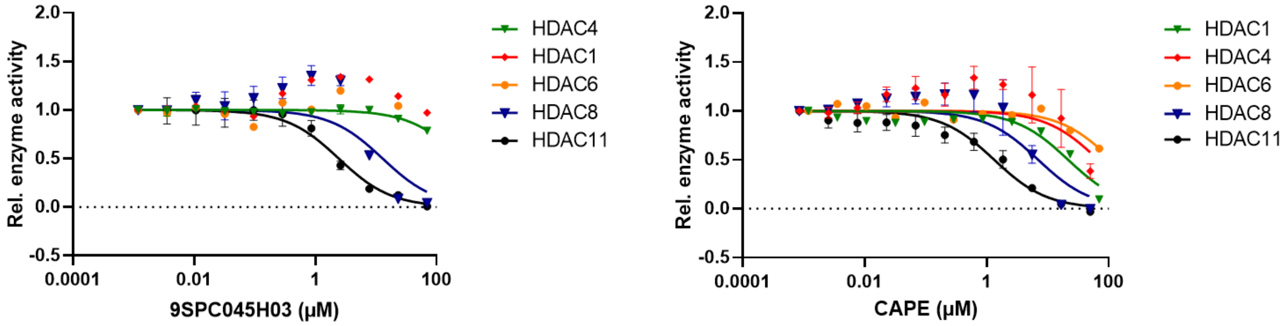

Figure S3: Dose-response curves of 9SPC045H03 and CAPE against a panel of HDAC isozymes.

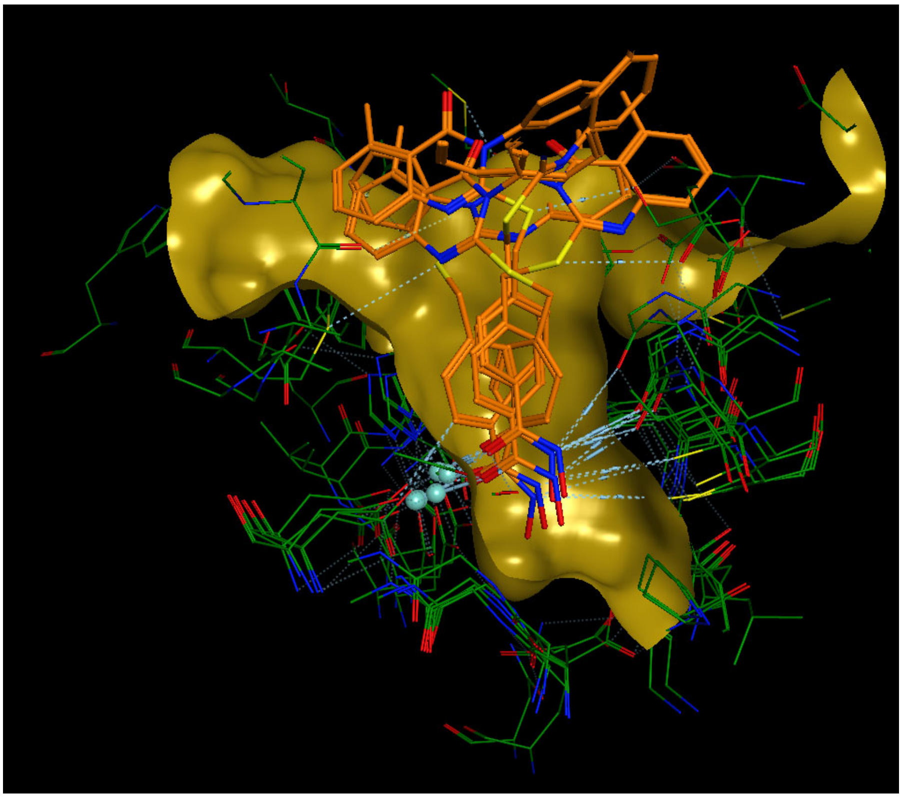

Figure S4: Superposed docking poses of cluster #1 hit 9HDA020C06 (orange) in complex with HDACs 1 (PDB-ID: 4BKX), 4 (PDB-ID: 4CBY), 6 (PDB-ID: 5EDU), 8 (PDB-ID: 3SFF) and 11 (AlphaFold structure) with very similar binding poses, particularly with respect to Zn^{2+}-coordination. The cyan spheres indicate the Zn^{2+}-ion and the ochre surface the conserved binding pocket.

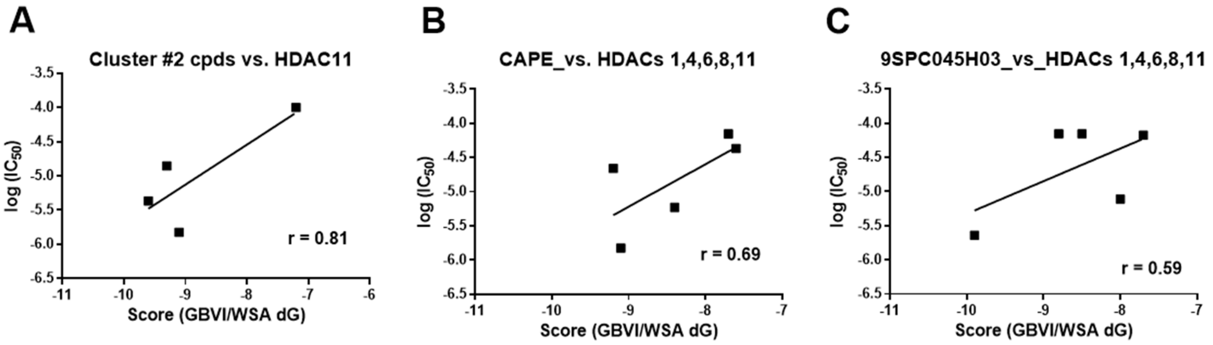

Figure S5: Correlation between docking scores and IC_{50}-values. Data correspond to A) compounds from cluster #2 inhibiting and binding to HDAC11 (Table S4), B) CAPE, which has been docked into HDAC 1, 4, 6, 8, and 11 (Table S5), and C) 9SPC045H03, which has has been docked into HDAC 1, 4, 6, 8, and 11 (Table S6). The Pearson correlation coefficient is shown in the lower right corner.

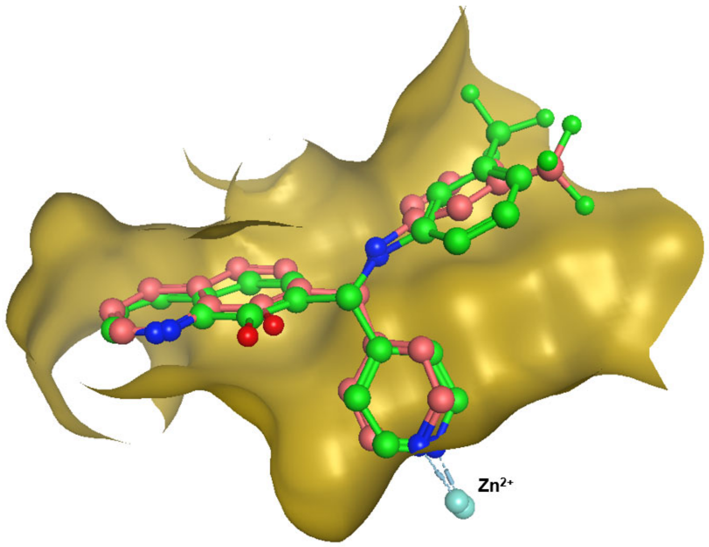

Figure S6: Overlay of similar binding poses of R- (green) and S- (dark pink) enantiomer of 9SPC045H03

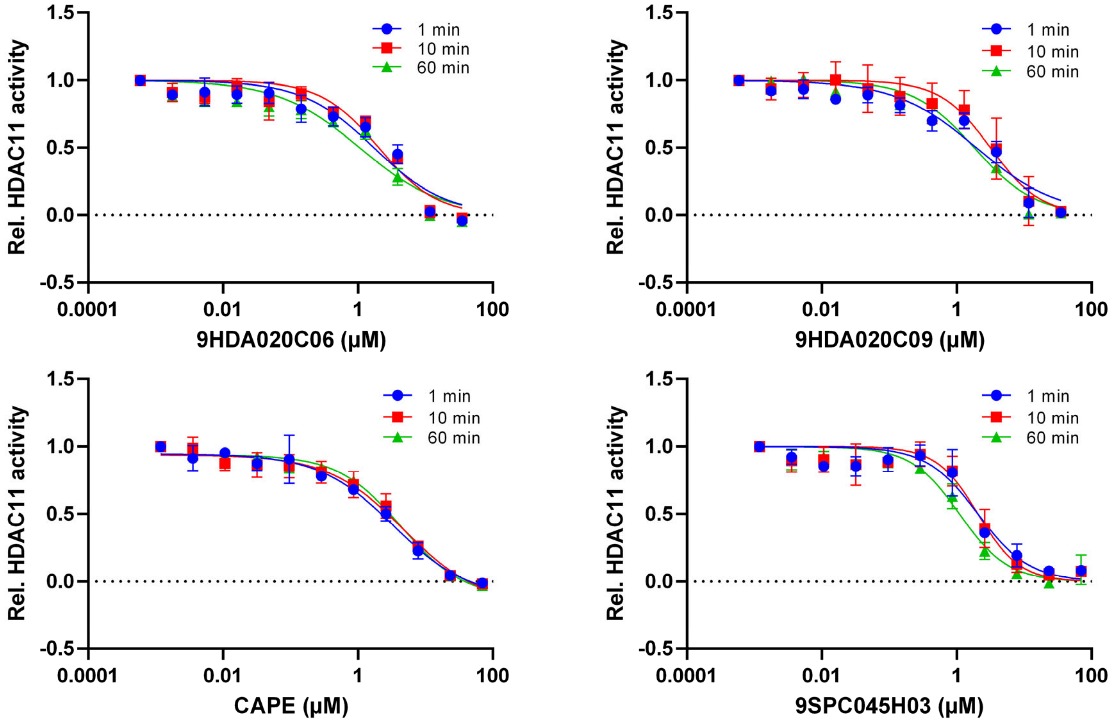

Figure S7: Timedependent dose-response curves of indicated compounds. Data points are shown as means with standard deviations (n=3).

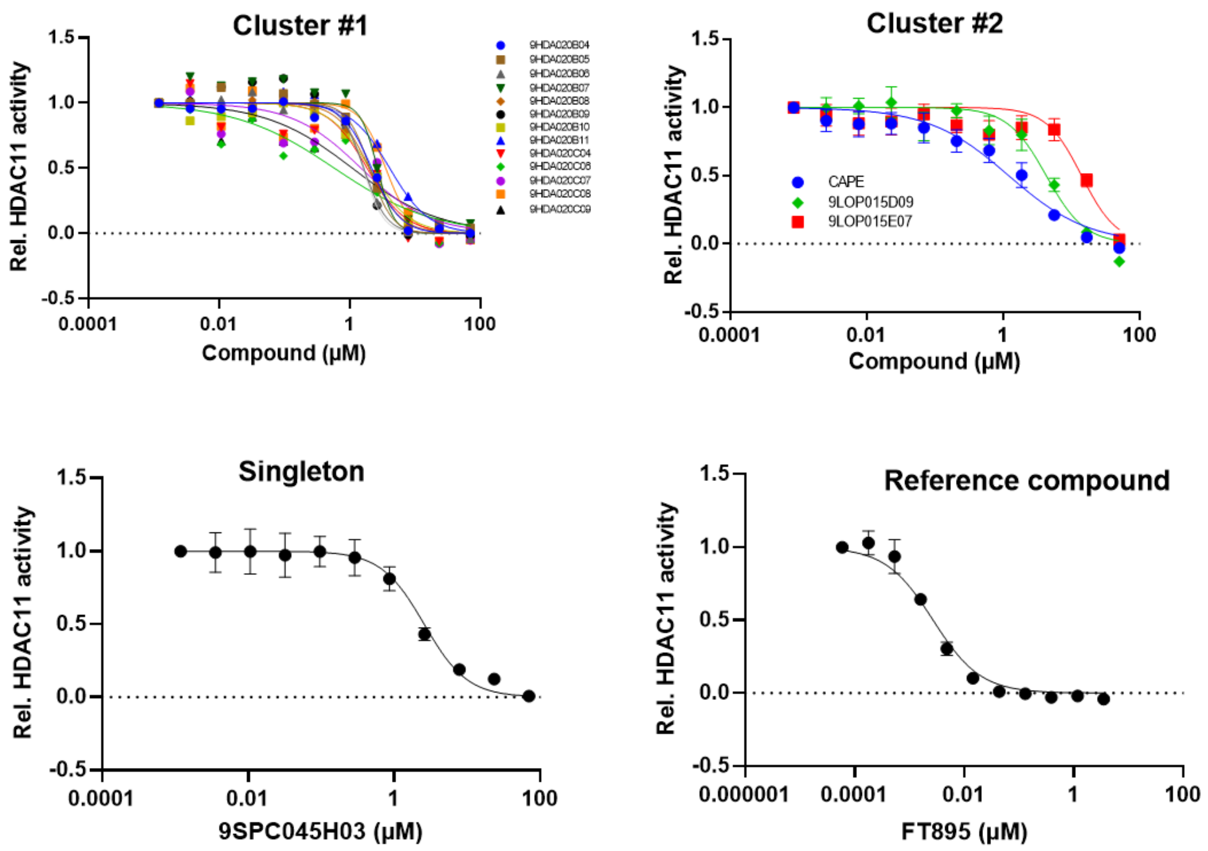

Figure S8: Dose-response curves of potent hit-compounds against HDAC11. Data points are shown as means with standard deviations (n=3).

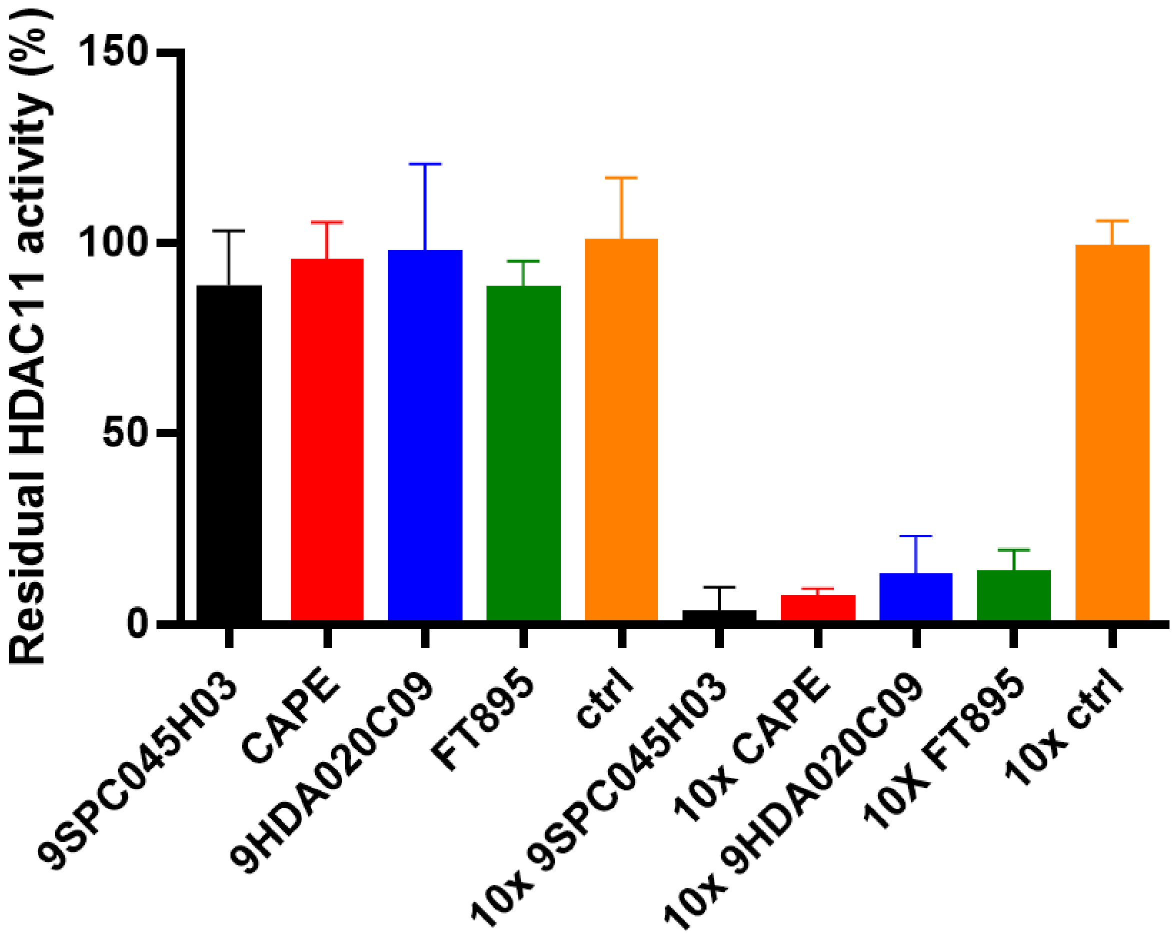

Figure S9: Rapid dilution experiments to demonstrate the reversibility of binding of the indicated compounds to HDAC11. The control experiments (orange) contain all assay reagents but no inhibitor. In the presence of 10-fold compound concentration, HDAC11 is still inhibited (see columns on the right). After rapid dilution, the compounds dissociate from the enzyme and enzyme activity is recovered (see columns on the left).

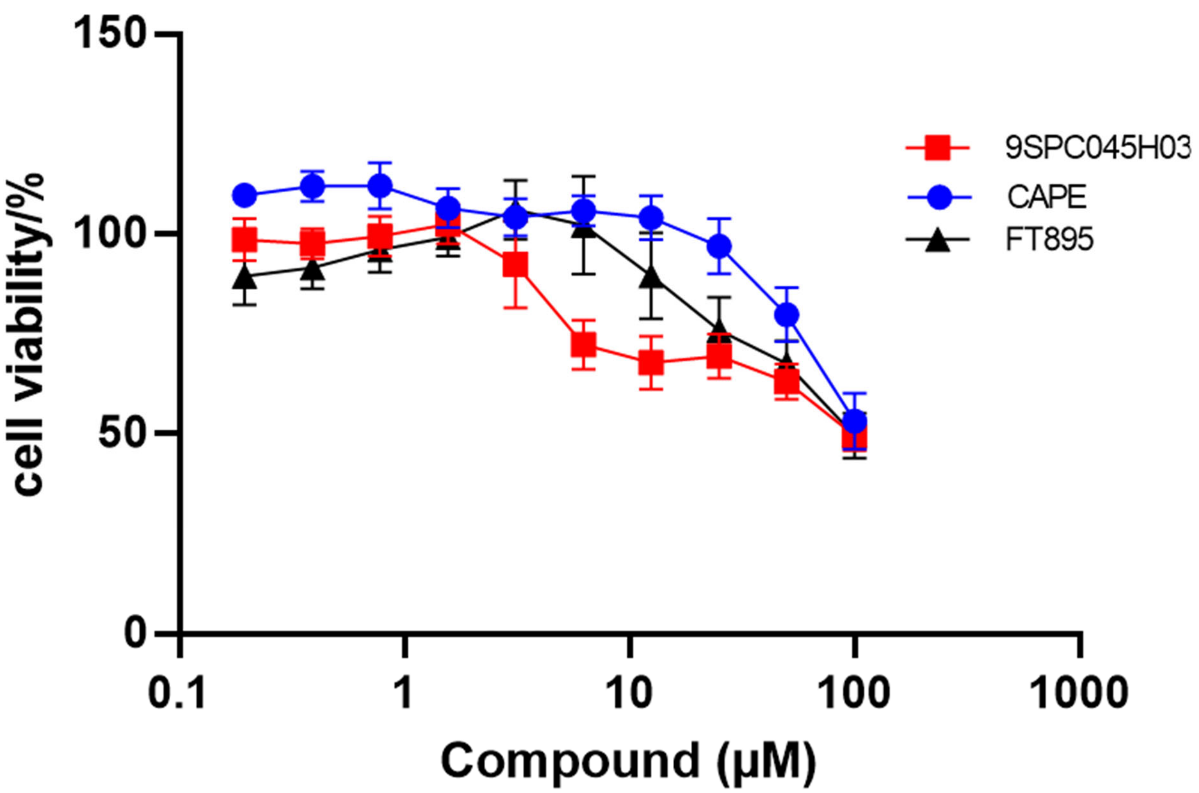

Figure S10: Cytotoxicity testing of indicated compound against HEK293 cell. 5000 HEK293 cells were seeded in 96-Well plates. After 48h Inkubationszeit at 37°C and 5% CO_2 in DMEM supplemented with 10% FBS and 1% Pen/Strep. A serial 1:2 dilution starting with 100 uM of compounds was added and incubated for a further 24h. XTT reagent was added and incubated for 4h. Afterwards the absorbance was measure at 450 nm and 600 nm. Data points are mean values ± standard error, n ≥ 6.

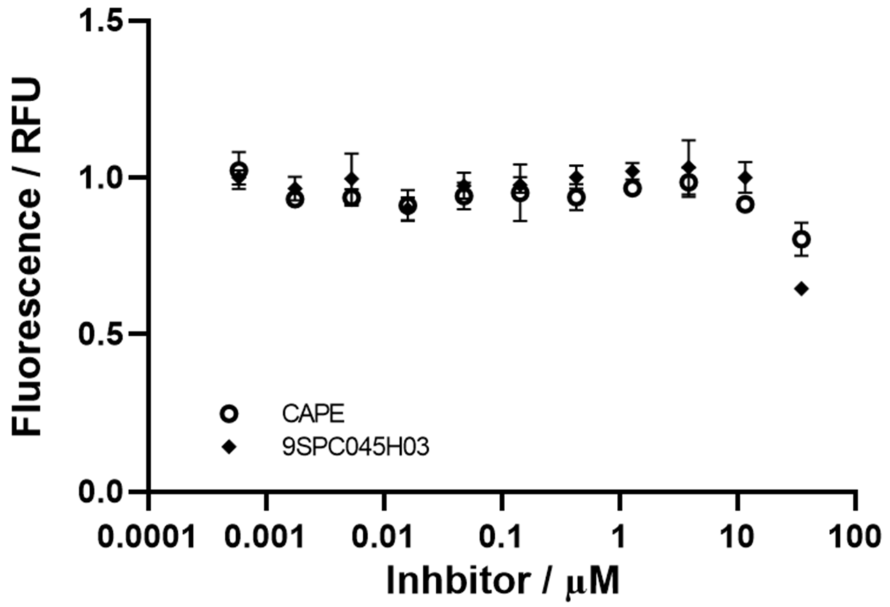

Figure S11: Control experiment to demonstrate that the inhibitory effect of CAPE and 9SPC045H03 in the enzyme activity assay is not due to compound-substrate interference. The dose-response curve is prepared as described for the enzyme activity assay in the

methods section in the manuscript, with the exception that the enzyme HDAC11 is omitted. The data are means and standard deviations, n = 3.

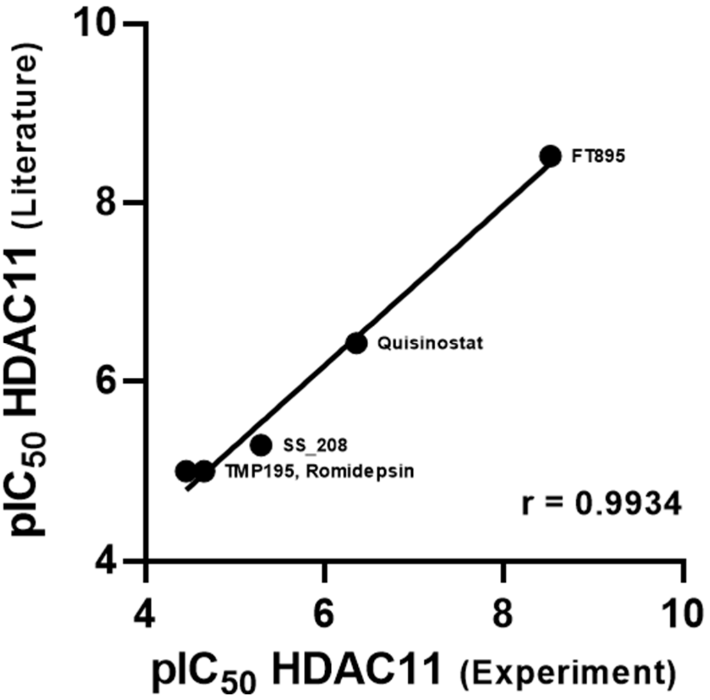

Figure S12: The negative decadic logarithm of published IC_{50}-values for the indicated HDAC11 inhibitors is plotted against the corresponding values determined with our enzyme activity assay. The data show a very high correlation with a Pearson coefficient of r = 0.9934.
